# Inclusion Complexation of Native and Functionalized α‑, β‑, and γ‑Cyclodextrins with PFAS: An Experimental and Molecular Simulation Study

**DOI:** 10.1021/acs.jpcb.6c02825

**Published:** 2026-07-18

**Authors:** Bowen Sha, Akhilesh Soodan, Kim Maren Lompe, Gokhan Barin, Thijs J. H. Vlugt, Loukas D. Peristeras, Othonas A. Moultos

**Affiliations:** † Engineering Thermodynamics, Process & Energy Department, Faculty of Mechanical Engineering, 2860 Delft University of Technology, Leeghwaterstraat 39, Delft 2628 CB, The Netherlands; ‡ Sanitary Engineering, Water Management Department, Faculty of Civil Engineering and Geosciences, 2860 Delft University of Technology, Stevinweg 1, Delft 2628 CN, The Netherlands; § 691797 Cyclopure, Inc., Evanston, Illinois 60201, United States; ∥ Molecular Thermodynamics and Modeling of Materials Laboratory, Institute of Nanoscience and Nanotechnology, National Center for Scientific Research “Demokritos”, Aghia Paraskevi, Attikis GR-15310, Greece

## Abstract

β-Cyclodextrin (β-CD)-based polymers have shown high adsorption capacities for removing per- and polyfluoroalkyl substances (PFAS) from drinking water. PFAS capture by these materials involves many physical and chemical processes, such as adsorption and inclusion complexation. Quantifying the underlying host–guest binding between CDs and PFAS nevertheless remains essential because it governs the primary inclusion step. Here, we investigated native and linker-modified CD–PFAS inclusion complexes in aqueous solution using isothermal titration calorimetry (ITC) and molecular dynamics (MD) simulations with the attach–pull–release (APR) method. We computed the Gibbs free energies of binding for α-, β-, and γ-CDs with seven linear PFAS, including perfluorocarboxylic acids and perfluorosulfonic acids, and found reasonable agreement with our experimental measurements and previously reported data. Comparison between implicit and explicit solvent calculations suggests that the apparently better agreement of the implicit solvent model for some native monomer complexes is likely due to error cancellation rather than a more transferable physical description, whereas explicit solvent is required to capture solvent-related salt and linker effects. Overall, β-CD exhibited the strongest binding affinities, whereas α-CD showed negligible affinities and γ-CD bound PFAS more weakly than β-CD. Hydrogen bonding, interaction-energy decomposition, and solvent-accessible surface area showed that host–guest hydrogen bonding cannot uniquely predict affinities, and that hydrophobic dehydration plays a dominant role in binding. We further examined how background ion concentration affects β-CD–PFAS binding and found that explicit solvent simulations capture a clear salt dependence, with Li/Merz ion parameters describing the high-salinity trend more reasonably than the Joung–Cheatham model. To mimic the local microenvironment surrounding CD units in polymers, we also examined three linker-modified β-CD models containing phenyl groups. Bind3P water correctly reproduced the experimentally reported enhancement of PFAS adsorption with increasing linker number, and energy decomposition showed that linker groups strengthen PFAS binding by enhancing local hydrophobic confinement and specific linker–guest interactions. Overall, this combined experimental and computational study provides molecular-level insight into the building blocks of cyclodextrin polymers and lays the groundwork for future in silico construction of CD polymer models for PFAS adsorption under diverse water-matrix conditions.

## Graphical abstract

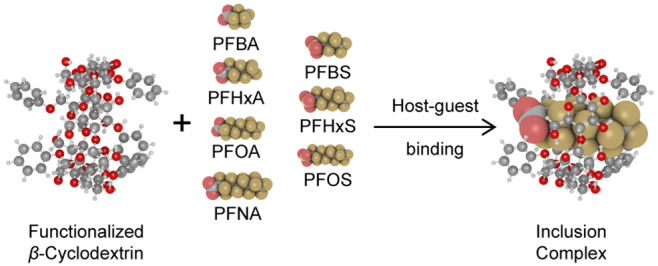

## Introduction

Per- and polyfluoroalkyl substances (PFAS), also referred to as “forever chemicals,″^1^ are defined by the Organisation for Economic Co-operation and Development^2^ (OECD) as fluorinated substances containing at least one fully fluorinated methyl or methylene carbon atom (i.e., substances containing at least one perfluorinated methyl group, −CF_3_, or one perfluorinated methylene group, −CF_2_−). The physicochemical behavior of PFAS depends not only on chemistry, but also on chain length, i.e., longer-chain PFAS are generally more hydrophobic, whereas shorter-chain PFAS are comparatively more hydrophilic.^3,4^ Many PFAS also show strong oleophobicity and high thermal stability.^5–9^ Such properties are important in applications such as nonstick coatings^10,11^ firefighting foams^10,12,13^ and other applications.^14–16^ The high stability conferred by the strong carbon–fluorine (C–F) bonds^17–19^ makes PFAS highly persistent in the environment and resistant to biodegradation. PFAS can penetrate the human body through skin contact^20,21^ food intake^22,23^ and other pathways, potentially inducing reproductive toxicity^24–26^ kidney damage^27–29^ endocrine disruption^30–32^ and other adverse health effects.^33,34^ While studies on the full extent of PFAS-related biological hazards are still ongoing, PFAS removal from water remains essential. For example, the Dutch National Institute for Public Health and the Environment (RIVM) has proposed a benchmark of 4.4 ng PFOA-equivalents/L for 38 PFAS (not a total PFAS concentration), and a separate threshold of 2.2 μg/L for trifluoroacetic acid (TFA) when no other PFAS are present.^35^

Several PFAS treatment technologies have been developed, including electrochemical oxidation^36–39^ catalytic decomposition^39–41^ and adsorption.^42,43^ Adsorption stands out as a highly promising approach due to operational simplicity, relatively low cost, and flexibility in adsorbent selection.^44–48^ Currently, various porous materials are used for PFAS removal, among which activated carbon (AC) has been particularly widely adopted due to its high surface area and strong hydrophobic interactions with PFAS.^49–51^ As a conventional adsorbent, typical AC in water treatment has a specific porosity of ca. 1000 m^2^/g^51,52^ and its hydrophobic pores can enhance interactions with PFAS. Zeolites^53–55^ and metal–organic frameworks^56–58^ (MOFs) are promising candidates that are currently investigated, but are still at low technology readiness level. Although the aqueous stability of different MOFs varies and has been discussed extensively, many studies have reported that MOFs used for PFAS removal are not sufficiently stable in water.^57,59–61^ In many cases, current porous materials show lower adsorption affinity for short-chain PFAS than for long-chain variants (partly due to low hydrophobicity and fast diffusion of short-chain PFAS), resulting in poor removal efficiency.

β-Cyclodextrin (β-CD) is a promising building block for porous polymer materials in water treatment.^62^ It is a low-cost, nontoxic, naturally occurring cyclic oligosaccharide derived from starch^63,64^ composed of seven glucose units forming a ring-shaped cavity. The exterior of the cavity is relatively polar, while the interior is nonpolar, enabling β-CD to form stable inclusion complexes with various compounds of suitable size, shape, and polarity. Unlike AC, which relies primarily on nonspecific hydrophobic adsorption, β-CD-based polymers offer more selective PFAS capture through size- and shape-complementary host–guest interactions, representing an emerging class of adsorbents with significant potential for targeted PFAS remediation. Porous polymers constructed from CDs offer a promising platform for PFAS removal, owing to their structural tunability,^65^ physicochemical stability,^66^ and relatively simple synthesis pathways.^67,68^ In recent years, numerous β-CD-based polymers with strong PFAS removal capabilities have been synthesized,^65,69,70^ These materials can capture diverse PFAS molecules through mechanisms such as inclusion complexation, hydrogen bonding, electrostatic interactions, and hydrophobic effects.^7,62–64,71^

A key prerequisite for mechanistic understanding of CD–PFAS inclusion is a reliable description of CD conformational fluctuations and cavity hydration. Sandilya et al.^72^ analyzed the conformations and hydration of native α-, β-, and γ-CDs in water and computed the number and energetics of water molecules in the cavity to study how dehydration contributes to molecular recognition. Chemical modifications can further alter CD flexibility and interaction patterns. For example, Geue et al.^73^ showed that methylation perturbs the intramolecular hydrogen-bond network of CD, and can strengthen host–guest interactions through a combination of increased host flexibility and enhanced intermolecular hydrogen bonding. Moreover, thermodynamic binding parameters from experiments can provide useful points of comparison for molecular modeling. As an example, Liu et al.^74^ combined calorimetric/structural characterization with conformational search and quantum chemical simulations to quantify β-CD host–guest binding with oleic acid. For predictive modeling of CD host–guest thermodynamics, robust free energy methods are required. Henriksen et al.^75^ developed the Attach–Pull–Release (APR) method to compute absolute binding free energies for host–guest systems, including β-CD. Erdös et al.^76^ applied the APR method to a wide range of organic micropollutants complexing with β-CD, and reported in close agreement with experiments, suggesting that APR is well suited for a systematic study of CD inclusion complexes.

Despite the growing use of CD-based polymers for PFAS removal, systematic molecular-level thermodynamic characterizations of CD–PFAS inclusion complexes remain limited. The binding is generally understood to be driven primarily by hydrophobic interactions, with electrostatic and other interactions playing a secondary role, yet the relative magnitudes of these contributions have not been quantified. Although polymerization is known to influence PFAS uptake, the energetic contributions of linker groups to the inclusion thermodynamics remain largely unknown. We therefore focused on monomeric CDs as well-defined molecular models that capture the key inclusion-complexation step underlying PFAS uptake by CD-based polymer adsorbents. In this study, we combined the APR method with isothermal titration calorimetry to investigate host–guest binding between native α-, β-, and γ-CDs with seven linear PFAS (PFCAs and PFSAs, see Figure 1a). These target compounds were selected from PFAS included in the RIVM relative potency factor framework, which provides a policy-relevant basis for focusing on substances of recognized environmental concern.^77^ The set also spans representative chain lengths and both carboxylate and sulfonate headgroup chemistries, enabling a systematic comparison of how molecular size and headgroup identity affect inclusion thermodynamics. Figure 1b shows the structures and cavity sizes of the three CDs, and Figure 1c defines the two initial host–guest binding orientations considered in the simulations, with the anionic headgroup directed toward either the primary or secondary hydroxyl rim. Additionally, to better mimic the local chemical environment in CD-based polymer adsorbents, we studied linker-modified β-CD derivatives with 1, 3, or 5 phenyl substituents. These derivatives allow us to investigate how linker-like groups influence PFAS binding.

**Figure 1:**
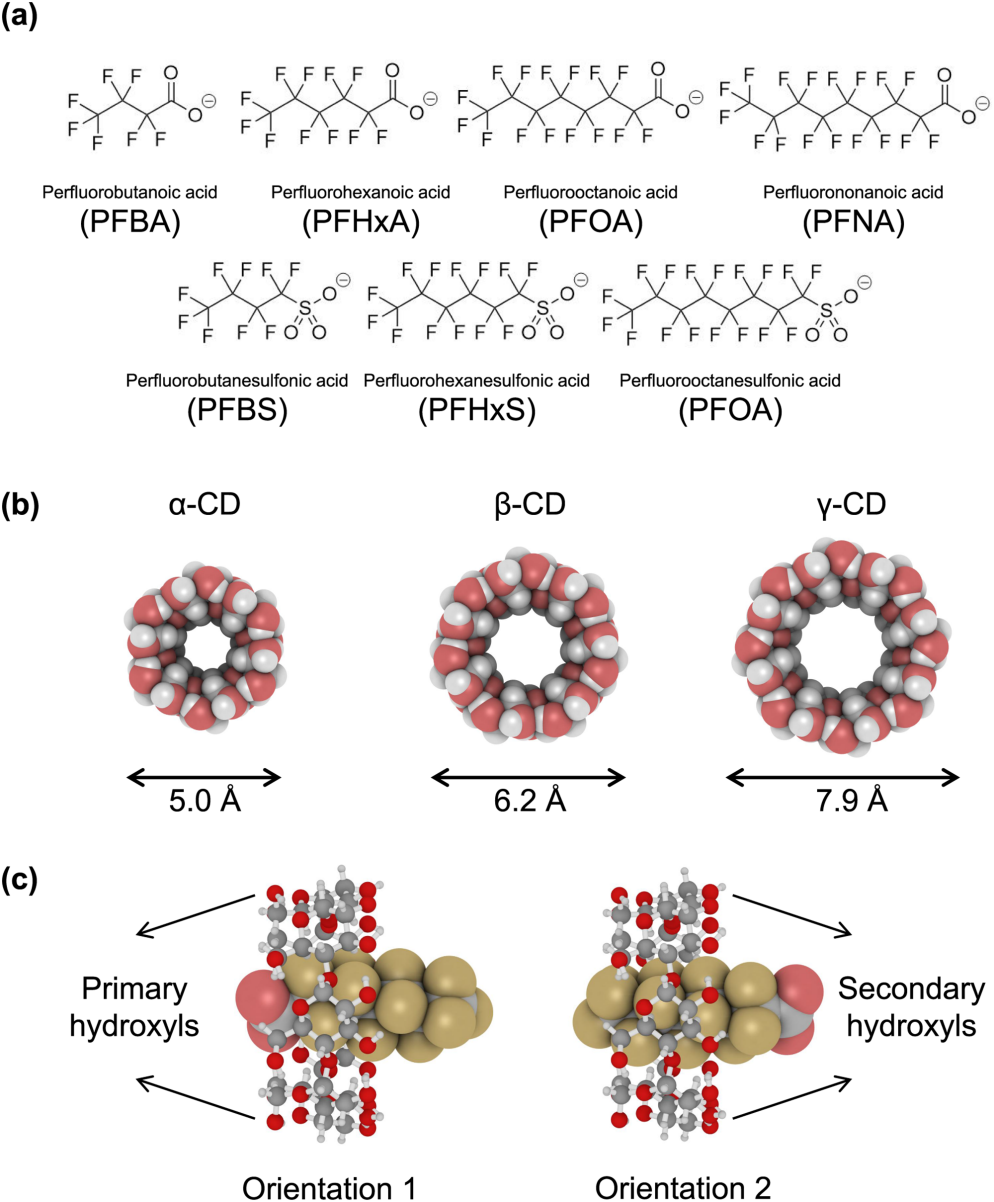
(a) Chemical structures of the seven PFAS studied (shown in their anionic form); the first row shows perfluorocarboxylic acids (PFCAs), and the second row shows perfluorosulfonic acids (PFSAs). (b) Top views of α-, β-, and γ-CD with approximate cavity diameters. (c) Definition of the two initial host–guest orientations used in the simulations. Molecular renderings were generated using iRASPA.^80^

The ionic strength of water strongly affects PFAS behaviors, and therefore, the adsorption capabilities of CD-based adsorbents.^5,65,78,79^ At the molecular level, increasing NaCl concentration lowers the self-diffusion coefficients of native CDs in aqueous solution, which has been attributed to preferential interactions of Na^+^ ions with CD hydroxyl groups together with an increase in solvent viscosity.^78^ Dissolved salts also change the solution behavior of PFAS. Higher ionic strength promotes PFAS aggregation and interfacial activity through salting out,^79^ while increasing cation concentration strengthens the adsorption of long-chain anionic PFAS by screening the electrostatic repulsion between PFAS head groups and thereby enhancing hydrophobic tail interactions.^5^ At the materials level, experiments with porous β-CD polymer adsorbents show that salt solutions mainly suppress the removal of short-chain PFAS, which depend more strongly on electrostatic interactions, whereas the removal of long-chain PFAS is much less affected.^65^ However, it is still unclear how the NaCl concentration changes the host–guest binding free energy between β-CD and PFAS at the molecular level, and this question motivates the present study.

By combining ITC measurements with APR calculations, we conducted a systematic thermodynamic analysis of host–guest binding for multiple cyclodextrin variants with a series of linear PFAS. Beyond reporting binding free energies, we use interaction-energy decomposition and hydrogen-bond analyses to clarify the molecular interactions that govern CD–PFAS complexation. We further examine how the NaCl concentration changes the binding free energy of the β-CD–PFOA complex, which provides molecular-level insight into the effect of background ions on PFAS removal by CD-based adsorbents at saline conditions. By replacing CD hydroxyl groups with linkers used in polymeric materials, we assess how local chemical modification alters the energetics of PFAS binding. In addition, performing simulations using implicit and explicit solvent models reveals that full atomistic representation is not necessary for accurately predicting binding energies; nevertheless, explicit solvent is required to describe solvent-mediated physical mechanisms that control binding (e.g., hydrogen bonding). Our findings establish a detailed molecular picture of CD–PFAS recognition across different application-relevant scenarios, support the use of molecular simulation for the design of PFAS-binding cyclodextrin materials, and provide a basis for future in silico modeling of CD-based polymer adsorbents. Overall, our study shows that β-CD is the most favorable native host for the diverse PFAS species considered here, whereas α-CD and γ-CD bind weakly with the guest molecules. Our results indicate that hydrophobic effects, rather than host–guest hydrogen bonding alone, are the main thermodynamic driving force for binding, and that explicit solvent simulations more reliably capture the effects of salt and linker modification, which are relevant to cyclodextrin-based polymer adsorbents.

The remainder of this manuscript is organized as follows. In the Methods section, we describe the simulation methodology and computational details, including the APR-based free energy framework, force field and solvent/ion models, and the ITC protocols used for experimental thermodynamic characterization. In Results and Discussion, we present the computed and measured host–guest thermodynamics, together with hydrogen bond and energy decomposition analyses, for native and linker-modified CD systems under different binding orientations and ionic strengths. The main conclusions and implications for the molecular design of CD-based PFAS adsorbents are then summarized in the Conclusions section. Supporting information includes scripts and parameter files in AMBER format for the PFOA−β-CD system, together with tables reporting the ITC results for α-, β-, and γ-CD with selected PFAS and the simulation-derived binding free energies for the native CD systems and for β-CD with 2,4,6-trichlorophenol and hexanoate.

## Methods

### Molecular Simulations

All simulations were performed using the pAPRika software package with AMBER25.^81^ As described in detail in the original pAPRika paper,^75^ this program implements the APR method, which primarily calculates the work required to pull the guest molecule out of the host cavity, thereby providing a method for computing the binding free energy of the host–guest complex. The Q4MD-CD force field and corresponding atomic partial charges^82^ were applied to α-, β-, and γ-CDs. This force field, developed by combining the GLYCAM^83^ and AMBER99SB^84,85^ reproduces both structural and dynamic properties of CDs and provides a reliable description of CD hydrogen bonding.^82^ For all PFAS molecules, the Generalized Amber Force Field^86^ (GAFF) was used. This choice is motivated by two considerations. First, GAFF was developed for organic small molecules, a category that includes the PFAS considered here. Second, the Q4MD-CD force field used for cyclodextrins was derived from the AMBER force field family, so a high compatibility with the original GAFF parameters is expected. Different studies also reported using GAFF for simulations of adsorption and binding of PFAS with various materials, such as clays,^87^ graphite^88,89^ covalent organic frameworks,^88^ and proteins^90,91^ showing good agreement with experimental data.

Here, we studied seven linear PFAS, comprising four PFCAs (PFBA, PFHxA, PFOA, and PFNA) and the corresponding PFSAs (PFBS, PFHxS, and PFOS), as shown in Figure 1a. These compounds are included in the RIVM relative potency factor framework and are considered to pose relatively high environmental concern,^77^ and therefore were selected in our study. All PFAS were modeled in their deprotonated forms, consistent with the generally strong acidity of PFCAs and PFSAs,^92^ which are therefore typically present as anions in aqueous solution. We also considered linker-modified β-CD systems in which phenyl groups were introduced by replacing hydroxyl hydrogen atoms on the primary and/or secondary rims of the CD cavity. The 1-substituted β-CD has a single phenyl group on a secondary hydroxyl site, the 3-substituted β-CD has one phenyl group on a primary hydroxyl site and two on secondary hydroxyl sites, and the 5-substituted β-CD contains two phenyl groups on primary hydroxyl sites and three on secondary hydroxyl sites. The atomic charges for PFAS and phenyl were derived using the RESP 2_0.5_ method^93^ calculated by Multiwfn^94,95^ program, based on the B3LYP/def2-TZVP level of theory with molecular structures optimized at the B3LYP/6–31G* level using Gaussian 09 Rev B.01.^96^ Compared to the original RESP scheme, RESP 2_0.5_ provides a more physically rigorous fixed-charge description by incorporating solvent-induced molecular polarization through a balanced combination of gas-phase and aqueous-phase electrostatic information.^93^ In addition, the effectiveness of RESP-based charge models in APR calculations for cyclodextrin host–guest systems has been demonstrated in previous studies by Henriksen et al.^75^ and Erdös et al.^76^ Given both this stronger physical basis and the proven performance of such charge models in related CD inclusion studies, we adopted RESP 2_0.5_ charges here. For phenyl-modified β-CDs, missing force field parameters were taken from GAFF.^86^ The Bind3P explicit solvent model was used for water molecules. Building upon the widely used TIP3P model, Bind3P has been specifically optimized for host–guest complex binding free energy calculations.^97^ While performing well in reproducing the liquid properties of pure water, Bind3P water model significantly reduces errors in free energy calculations for host–guest systems compared to experimental values.

To account for the effect of salinity on CD–PFAS binding, we considered four NaCl concentrations (0, 1, 2, and 3 mol NaCl/1 kg H_2_O). Because no ion force field has yet been explicitly parametrized for the Bind3P water model, and Bind3P is a modified variant of TIP3P, we adopted ion parameters developed for TIP3P. Specifically, we used the Joung–Cheatham^98^ (JC) and the Li/Merz^99^ (LM) ion models. Unlike other commonly used ion force fields (e.g., Madrid,^100^ JC and LM ion models use nonscaled charges on the ions. For Li/Merz, two parameter sets are available that are fitted to hydration free energies (HFE) and ion–oxygen distances (IOD), respectively. Both sets were tested in this study.

Figure 1c shows the two initial host–guest binding orientations used in the simulations: Orientation 1, with the anionic headgroup pointing toward the primary hydroxyl rim, and Orientation 2, with the headgroup pointing toward the secondary hydroxyl rim. In this work, the primary rim refers to the side of the CD cavity with primary hydroxyl groups, whereas the secondary rim refers to the side with secondary hydroxyl groups. To compare with the explicit solvent calculations, we also performed simulations of the host–guest binding free energies using a Generalized Born^101^ implicit solvent model. In this approach, solvent molecules are not represented explicitly. Instead, a cavity is constructed around the solute, and the solvent effect is approximated as an external reaction field describing solute–solvent interactions. Beyond reducing the number of atoms in the simulation box, the absence of explicit solvent relaxation allows the system to reach equilibrium rapidly.

For explicit solvent simulations, a 2 fs time step was used with periodic boundary conditions (PBC) applied in all directions. Electrostatic interactions were calculated using the Particle Mesh Ewald (PME) summation method^102,103^ while nonbonded interactions were treated with a 0.9 nm cutoff distance. The pressure was set equal to 1 bar using a Monte Carlo barostat.^104^ The host–guest system was solvated in 3000 water molecules. In contrast, the implicit solvent simulations were performed without PBC and calculated the electrostatic interactions directly. Both explicit and implicit solvent simulations used the Langevin thermostat^105^ for controlling the temperature at 298.15 K, with the friction coefficient γ set to 1 ps^–1^, and the leapfrog algorithm for numerically integrating the equations of motion.

For all simulations, we followed the same three-step protocol. After creating the initial configuration, we minimized system energy using the steepest descent algorithm, and then we performed a 1 ns equilibration MD run in the *NPT* ensemble, followed by a 10 ns production run in the same ensemble. In pAPRika, the free energy work values along the attach–pull–release pathway were computed using the Multistate Bennett Acceptance Ratio (MBAR) approach, following Henriksen et al.^75^ Five independent parallel simulations were performed for each CD–PFAS combination, which were subsequently used to estimate both the binding free energies and their associated uncertainties.

Although two possible binding orientations were considered in the MD simulations, for convenient comparison with experiment, we computed an apparent binding free energy, Δ*G*_App_, using the following expression,

1 $$\Delta G_{\mathrm{App}}=-kT\ln\underset{i}{\sum }\exp\left(-\frac{\Delta G_{i}}{kT}\right)$$

where *i* denotes the different orientations considered and takes the values 1 and 2 in this study.

Postprocessing was performed on the production trajectories. The solvent-accessible surface area^106^ (SASA) change upon binding was computed as

2 $$\Delta \mathrm{SASA}={\mathrm{SASA}}_{\mathrm{CD}}+{\mathrm{SASA}}_{\mathrm{PFAS}}-{\mathrm{SASA}}_{\mathrm{Complex}}$$

where SASA_Complex_ denotes the SASA of the host–guest complex, and SASA_CD_ and SASA_PFAS_ correspond to the SASA of the host and guest in their unbound states, respectively. SASA, hydrogen-bond numbers, and the Lennard–Jones/Coulombic energy decomposition were carried out using GROMACS.^106–109^ In this work, a hydrogen bond was defined using a donor–acceptor (D–A) distance smaller than 0.35 nm and a H–D–A angle smaller than 30°, where D, A, and H denote the hydrogen-bond donor, acceptor, and hydrogen atom, respectively. Hydrogen-bond lifetimes were obtained from the time autocorrelation of a binary existence function (1 when a specific hydrogen bond is present and 0 otherwise), followed by averaging over all identified hydrogen bonds. Sample input files for free energy calculations are provided as Supporting Information.

The root-mean-square error (RMSE) and the mean relative deviation (MRD) of the host–guest binding free energies are calculated as follows to quantify the deviation between the computed and measured free energies:

3 $$\mathrm{RMSE}=\sqrt{\frac{1}{n}\overset{n}{\underset{i=1}{\sum }}{\left(\Delta G_{\mathrm{Sim},i}-\Delta G_{\mathrm{Exp},i}\right)}^{2}}$$

4 $$\mathrm{MRD}=\frac{1}{n}\overset{n}{\underset{i=1}{\sum }}\left|\frac{\Delta G_{\mathrm{Sim},i}-\Delta G_{\mathrm{Exp},i}}{\Delta G_{\mathrm{Exp},i}}\right|$$

where Δ*G*_Sim,*i*_ and Δ*G*_Exp,*i*_ denote the simulation and experimental binding free energies of the *i*-th host–guest complex, respectively.

## Experiments

### Materials

Analytical-grade PFAS were obtained from Sigma-Aldrich (≥98% purity). Individual PFAS stock solutions were prepared in HPLC-grade methanol (Sigma-Aldrich, MeOH) at 1 g L^–1^. The stock solutions were used to prepare 200 mg L^–1^ working solutions (corresponding to PFNA ≈ 0.43 mM, PFOA ≈ 0.48 mM, PFOS ≈ 0.40 mM, PFHxA ≈ 0.64 mM, PFHxS ≈ 0.50 mM, PFBA ≈ 0.94 mM, PFBS ≈ 0.67 mM). Working solutions were prepared by first evaporating MeOH at room temperature, followed by redissolution of PFAS salts in ultrapure water (18.2 MΩ·cm, Millipore) with 10 mM sodium chloride (NaCl) as background electrolyte. To avoid PFAS micelle formation, PFAS concentrations in the working solutions were kept well below the reported critical micelle concentrations (8 mM and 25 mM for PFOS and PFOA, respectively).^110^ A 5 mM cyclodextrin solution (α-, β-, and γ-CDs, ≥97%, Sigma-Aldrich) was prepared in 10 mM NaCl. All glassware and syringes were rinsed sequentially with ultrapure water, methanol, and ultrapure water to minimize PFAS contamination before use.

### Isothermal Titration Calorimetry

ITC was performed using a VP-ITC microcalorimeter (Malvern Panalytical) with a 1.8 mL cell volume, a reference power of 5 μcal s^–1^, and at a constant temperature of 25.0 ± 0.1 °C. The device was thermally equilibrated for 1 h before the experiments. All solutions were degassed briefly under vacuum (ThermoVac, Malvern Panalytical) before performing titrations. PFAS solutions were filled into the sample cell (1 PFAS per titration), and 300 μL of CD solution was loaded in the titration syringe. The titration consisted of 22 injections, with the first two injections of 2 μL being discarded. The remaining 20 injections were 10 μL each. Injections were spaced at 300 s; the filter period was 2 s; the injection lasted 10 s; and the stirring speed was set to 307 rpm.

Thermodynamic parameters were determined by fitting the integrated heat measurements to the 1:1 binding model (*K*_1:1_). ITC provides measurements of *K*, *n*, Δ*H*, and Δ*S* ^111,112^ from which Δ*G* can be calculated as

5 $$\Delta G=-RT\ln\left(\frac{K}{K^{◦}}\right)$$

where *R* is the gas constant (1.987 cal mol^–1^ K^–1^), *T* is the absolute temperature (K), *K* is the binding constant obtained from ITC (M^–1^), and *K*° = 1 M^–1^ is the standard state binding constant.

The quality of the ITC results was assessed using the *c*-value, defined as

6 c=nK[M]

where *n* is the stoichiometry (assumed to be 1 for the 1:1 CD–PFAS inclusion-complexation model), and [*M*] is the number of moles. For an accurate estimation of the binding constant *K*, the *c*-value should range between 1 and 1000.^112^

## Results and Discussion

### Host–Guest Binding of β-CD with PFAS

Figure 2 shows the computed apparent Gibbs binding free energies for β-CD with PFAS (apparent binding free energies are defined as described in the Methods section). The corresponding numerical values are reported in Tables S1a–S1c (experimental values) and Tables S2a and S2b (simulation values). Red and blue denote binding free energies from simulations with implicit and explicit solvent models, respectively, yellow denotes the binding free energies obtained from our experiments, and the white shaded data represent literature values from different sources. For shorter PFCAs, the experimental host–guest binding free energy increases with carbon-chain length, with an incremental contribution of approximately 1 kcal mol^–1^ per added CF_2_ unit. This trend is consistent with binding being predominantly driven by PFAS–CD van der Waals interactions, as discussed in more detail later in this section. Once the chain reaches the length of PFOS, however, the experimental binding free energy no longer increases, suggesting that the guest size approaches or exceeds the effective accommodation range of the β-CD cavity. Overall, the computed trends are in reasonable agreement with experiment and literature data, as shown in Figure 2, but the prediction for binding free energies of longer PFAS is less accurate, which likely contributes to the larger deviations observed for PFOS and PFNA. This suggests that the larger deviations for PFAS may be related not only to solvent treatment, but also to limitations of GAFF in describing the unique electronic environment of perfluoroalkyl chains^113,114^ for which dedicated force field development remains an active area of research.

**Figure 2:**
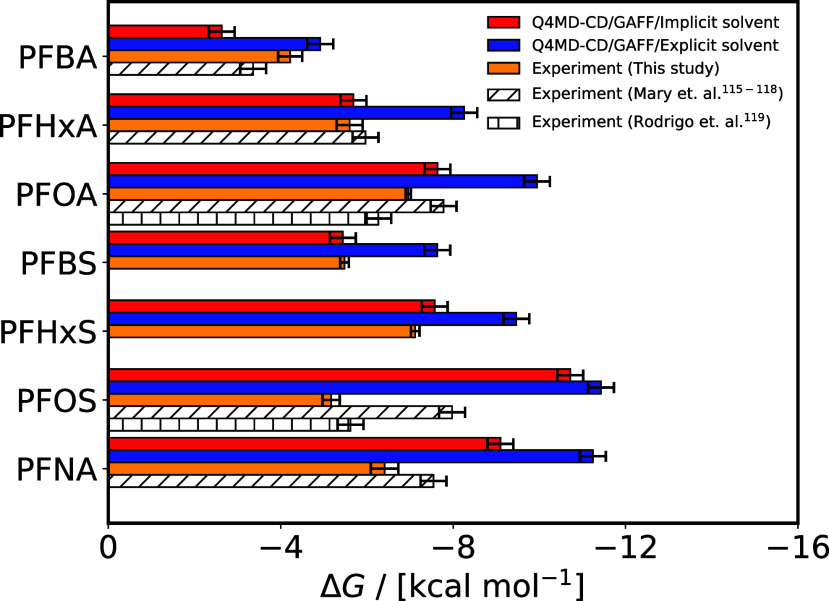
Gibbs free energies of host–guest binding between β-CD and different PFAS.^115–119^ The apparent free energies are used (eq 1) here to compare with experimental results.

Figure 2 suggests that the implicit solvent model is more accurate than the explicit solvent model (red and blue bars, respectively) for the β-CD-PFAS complexes. However, this deviation is not consistent across all computed CD-PFAS (i.e., 3 native CDs and 7 PFAS) binding energies. Over the full data set, the RMSE (eq 3) for the implicit and explicit solvent models are 3.2 and 3.3 kcal mol^–1^, respectively. Given that the uncertainty in our free-energy calculations is in the range of 0.2–0.4 kcal mol^–1^, no statistically significant difference between the two solvent models can be observed. In the previous studies by Erdös et al.^76^ and Yin et al.^97^ where the APR method and an explicit solvent model (i.e., the Bind3P force field) were used to predict the binding free energies of CDs with organic pollutants (modeled using GAFF), the reported RMSEs are equal to 0.9 and 1.7 kcal mol^–1^, respectively. These values may appear lower than the ones presented here, however, the absolute Δ*G* values in these studies are lower than ours, making a direct RMSE comparison less useful. To this end, we computed the MRDs (eq 4) using the data from the literature and from our study. The MRDs for α-CD and β-CD complexes with fatty acid salts with carbon chain lengths ranging from 4 to 8 from Yin et al.^97^ are 61.8% and 73.1%, respectively. Over our PFAS complexes, the corresponding MRDs for α-CD and β-CD are 64.2% and 53.7%, respectively. This comparison clearly shows that for β-CD-PFAS host–guest complexes, the Bind3P/GAFF atomistic models are a reasonable force field combination despite GAFF being a general-purpose model. Previous studies have already attempted to improve PFAS force fields based on GAFF^113^ or CGenFF.^120^ However, these efforts have mainly focused either on refining the description of perfluorinated carbon chain interactions to better reproduce condensed-phase equilibria,^120^ or on capturing PFAS–water interfacial properties.^113^ For such refinements to yield substantially improved descriptions of CD–PFAS host–guest interactions, further work is required toward this specific direction.

In Table S3 of the Supporting Information, we show a comparison between binding free energies for β-CD with hexanoate anion (HEX)^75^ and 2,4,6-trichlorophenol (TCP)^76^ computed both with explicit and implicit solvent. The binding free energies computed here using the explicit solvent model agree with the respective literature values within the statistical uncertainty (0.4–0.5 kcal mol^–1^). Meanwhile, the Δ*G* deviation for implicit solvent is 5-fold larger (ca. 2 kcal mol^–1^) for both systems compared to either our or literature computations using the explicit solvent. Interestingly, using implicit solvent, for TCP, the Δ*G* is overpredicted, while for HEX, it is underpredicted compared to the explicit solvent results, indicating a lack of consistency when the implicit model is used. Also based on the experimental values also shown in Table S3, simulations using the explicit solvent are very accurate for TCP (i.e., simulation: −3.2 kcal mol^–1^, experiment: −2.8 kcal mol^–1^), while for HEX, implicit model is closer to the measured value (i.e., simulation: −1.8 kcal mol^–1^, experiment: −2.3 kcal mol^–1^). These findings clearly indicate that a clear verdict on whether simulations with the implicit solvent are more accurate than the explicit solvent ones cannot be made based solely on the systems studied here.

It is important to note though that under the same GPU configuration, molecular simulations using the implicit solvent model are ca. 10–20 times faster than those with the explicit solvent. Also, for the same sampling length, the implicit solvent model yields substantially smaller statistical uncertainties in the calculated binding free energies. Another benefit of implicit solvent simulations is that the equilibration of our systems was reached after runs in the order of tens of picoseconds, while the explicit solvent required up to several hundreds of picoseconds. These advantages make calculations using the implicit solvent attractive for high-throughput calculations of CD-PFAS binding free energies which can be used for either qualitative studies and/or creating data sets for machine learning models.^121^

Because the explicit solvent model more realistically captures electrostatic and van der Waals interactions between solvent and solute molecules, and also better represents hydrogen-bonding interactions between CD/PFAS molecules and water, we focus on the explicit solvent results in the remainder of this work unless stated otherwise. To connect Δ*G*_Host–Guest_ with structural features of the CD–PFAS complexes, we used the SASA change upon binding, ΔSASA (see eq 2), as a simple structural descriptor. The linear regression between Δ*G*_Host–Guest_ and ΔSASA is shown in Figure 3 (with *R* ^2^ = 0.986 and RMSE = 0.2 kcal mol^–1^). Stronger binding free energies correspond to larger decreases in SASA, suggesting an important role of hydrophobic interactions in the host–guest binding.

**Figure 3:**
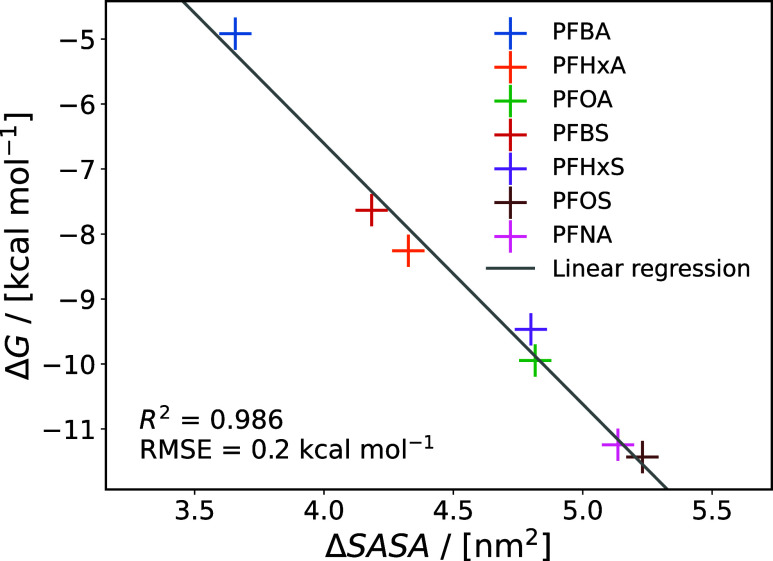
Correlation between binding free energy and desolvation descriptor for β-CD–PFAS complexes using the explicit solvent model. The apparent free energies are used here (eq 1).

Because cyclodextrins contain multiple hydroxyl groups and the PFAS molecules studied here carry hydrogen-bond-capable headgroups (carboxylate for PFCAs and sulfonate for PFSAs), hydrogen bonding is expected to influence host–guest association. To probe hydrogen bonds (H-bonds) between CDs and PFAS, we computed both the number and the lifetimes of hydrogen bonds in the fully bound host–guest complexes, in which the PFAS molecule remains completely included within the CD cavity without dissociating from it, and compared these results to that of free PFAS in water. Representative MD snapshots illustrating PFAS–CD and PFAS–water hydrogen bonds are shown in Figure 4. The results of H-bonds analysis are shown in Figure 5. Overall, the number of host–guest hydrogen bonds does not show a strong correlation with the binding free energy Δ*G*. In particular, for Orientation 2, β-CD forms almost no hydrogen bonds with PFAS. For all systems, the total PFAS-centered hydrogen-bond count changes very little upon complexation (typically within the statistical uncertainty). This indicates that complexation does not simply suppress PFAS–water hydrogen bonding; instead, the decrease in PFAS–water hydrogen bonds is partly compensated by formation of PFAS−β-CD hydrogen bonds. Figure 5 shows that Orientation 1 has more CD–PFAS hydrogen bonds than Orientation 2, consistent with stronger host–guest hydrogen bonding at the primary hydroxyl rim. In Orientation 1, however, both hydrogen-bond numbers and lifetimes vary only weakly across PFAS. Although PFSAs contain one additional oxygen atom relative to PFCAs, the total hydrogen-bond count increases only by a few tenths at most, which is not statistically significant. This behavior is consistent with the higher flexibility of primary hydroxyl groups, which can adapt to different PFAS headgroups with similar effectiveness. In Orientation 2, host–guest hydrogen-bond lifetimes are much shorter than in Orientation 1 and show a chain-length dependence. Because primary hydroxyl groups are attached to exocyclic carbon atoms on the side chains, these hydroxyl groups retain greater conformational flexibility. In contrast, secondary hydroxyl groups are attached to carbon atoms on the six-membered glucopyranose ring and are therefore more constrained by ring geometry. As a result, secondary hydroxyl groups are less flexible than primary hydroxyl groups, and sustained contact with PFAS is harder to maintain, especially for shorter PFAS that remain more deeply confined in the cavity and have fewer opportunities to interact with the secondary hydroxyls (see Figure 1c). In contrast, in Orientation 2, the higher-molecular-weight PFAS can still form occasional H-bonds with β-CD, which may be attributed to their greater chain flexibility. A longer perfluoroalkyl chain can more readily bend or fluctuate while remaining included in the cavity, thereby increasing the probability that the headgroup reaches the secondary hydroxyl rim. For PFAS–water hydrogen bonds, the lifetimes in the complex are slightly longer than for free PFAS in water, suggesting that confinement by the CD cavity can stabilize specific water contacts even though some PFAS–water contacts are sterically blocked upon complexation.

**Figure 4:**
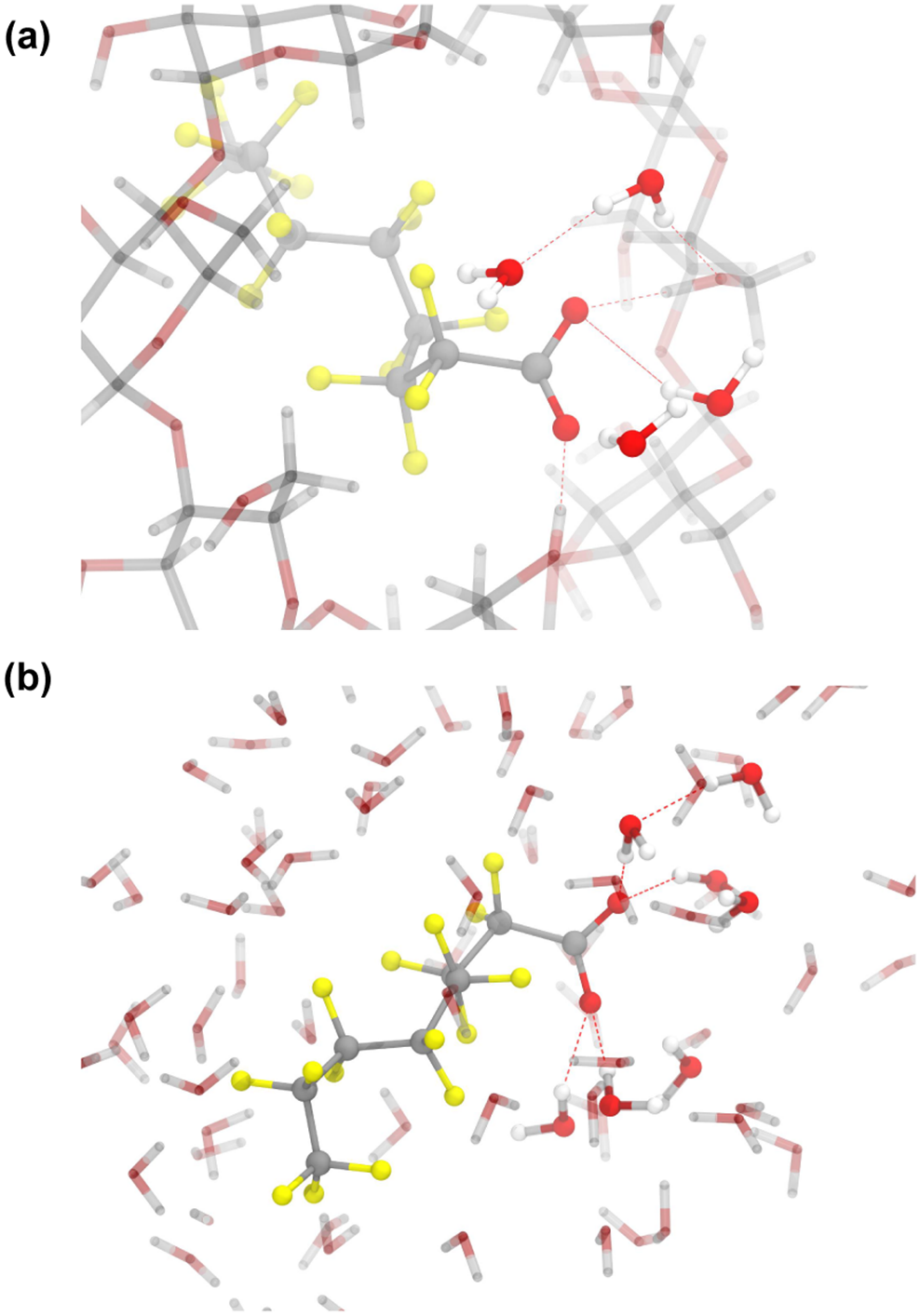
Representative hydrogen-bond configurations for PFOA in the β-CD system, showing hydrogen bonds formed between PFOA and β-CD in the inclusion complex (a) and between free PFOA and water molecules (b). White, gray, red, and yellow atoms represent hydrogen, carbon, oxygen, and fluorine, respectively.

**Figure 5:**
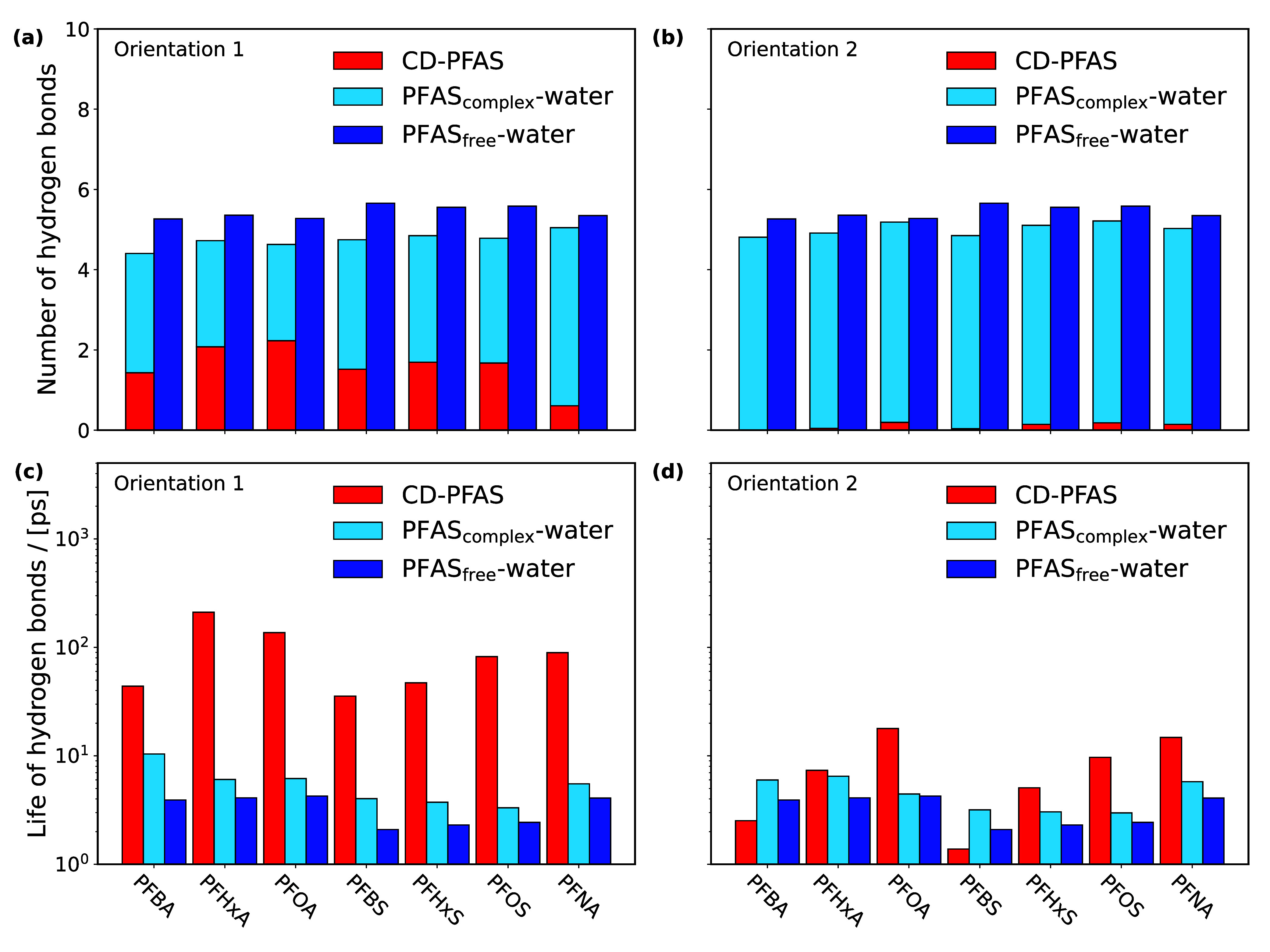
Number (a, b) and lifetime (c, d) of hydrogen bonds in β-CD–PFAS inclusion complexes for Orientation 1 (a, c) and Orientation 2 (b, d). PFAS_complex_ denotes PFAS in the inclusion complex, and PFAS_free_ denotes free PFAS in pure aqueous solution.

To analyze the interactions of the CD–PFAS complex in aqueous solution, including both the interactions within the host–guest complex and those with the surrounding solvent, we decomposed the interaction energies into Lennard–Jones (LJ) and Coulombic components. For comparison, we applied the same decomposition to free PFAS in water to assess solvation energetics. As shown in Figure 6, in Orientation 1, the CD–PFAS interaction contains a substantial Coulombic contribution for most PFAS, whereas in Orientation 2, the Coulombic term is uniformly small across all PFAS, and binding is dominated by the LJ term. For PFAS–water interactions, both in the complex and in the free state, the Coulombic component remains dominant with a smaller LJ contribution, and the LJ part increases with PFAS chain length.

**Figure 6:**
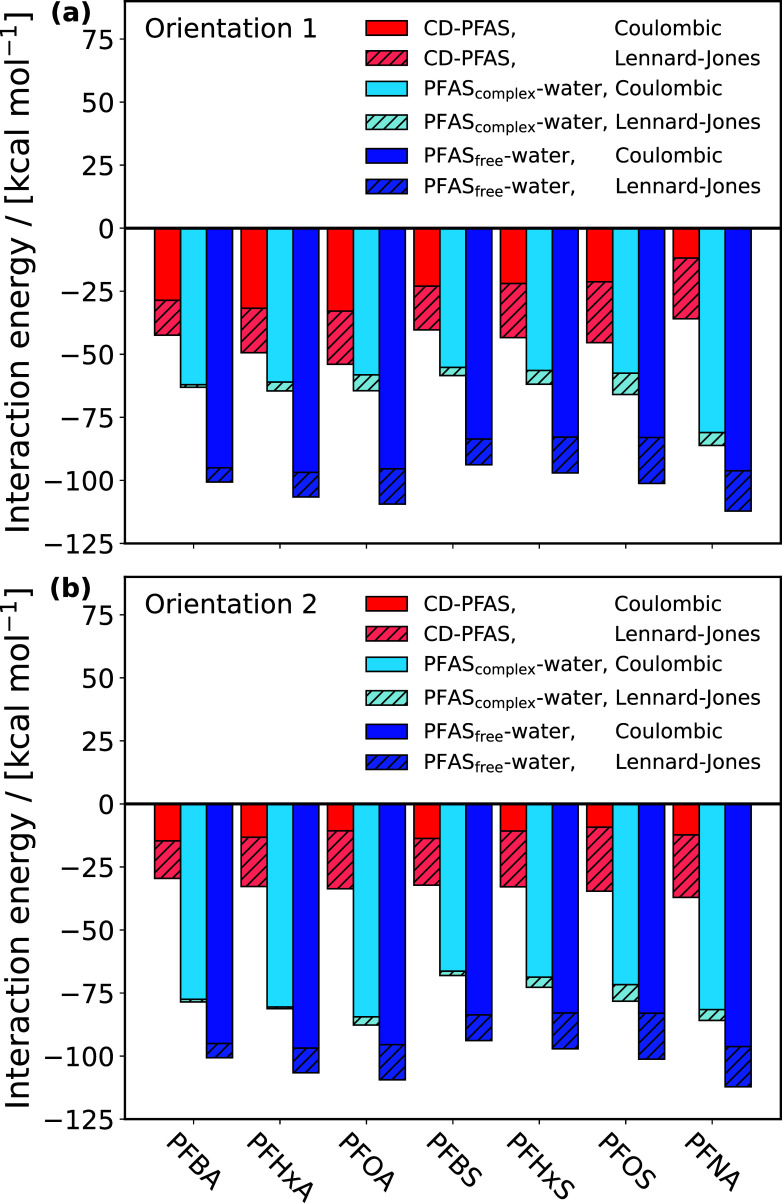
Energetic contributions in β-CD and PFAS systems for (a) Orientation 1 and (b) Orientation 2, where PFAS_complex_ denotes PFAS in the inclusion complex and PFAS_free_ denotes free PFAS in pure aqueous solution.

Taken together, results for the hydrogen-bond and energy-decomposition suggest a geometry-driven binding mechanism. Because the primary rim of β-CD is narrower, fewer water molecules can access this region. Therefore, binding is more favorable when the hydrophobic fluorocarbon tail is oriented toward the primary side, while the charged headgroup points toward the more hydrated secondary side. By contrast, when the charged headgroup is placed at the primary rim, hydrogen bonding with primary hydroxyls can provide local stabilization, but this orientation simultaneously exposes the hydrophobic tail to the wider opening on secondary side, where it contacts more water molecules, which is overall unfavorable for PFAS–CD binding.

### Host–Guest Binding of α- and γ-CD with PFAS

The corresponding numerical values of binding free energy are provided in Tables S2a and S2b (simulation results). These results show that PFAS binding to α- and γ-CD is generally weak, which makes estimating Δ*G*_Host–Guest_ for these hosts more challenging. Compared to β-CD, both α- and γ-CD exhibit substantially lower affinity for PFAS. In most cases, α-CD shows negligible binding, whereas γ-CD has only about less than half of the binding strengths of β-CD. This is consistent with cavity-size matching. The β-CD cavity offers a better fit for PFAS, enabling stronger hydrophobic contributions and more favorable LJ contacts. In contrast, the smaller cavity of α-CD restricts efficient host–guest packing, while the larger cavity of γ-CD reduces the quality of interfacial contacts, both of which weaken binding.

To facilitate a direct comparison across the three hosts, we selected PFOA as a representative guest, both for clarity of presentation and because PFOA is a commonly detected PFAS with well-documented and non-negligible health and environmental risks. As shown in Figure 7, α-CD forms more (and longer-lived) hydrogen bonds with PFOA, yet it still exhibits the weakest overall binding. This indicates that hydrogen bonding is not the primary determinant of affinity in these complexes. Although the hydrogen-bond metrics suggest stronger local CD–PFAS contacts for α-CD, these factors reflect only static structural features, whereas binding strength depends on the free energy profile associated with removing PFAS from the CD cavity. The smaller cavity of α-CD can enforce closer geometric contact between PFAS and the host at certain configurations, but this geometric confinement does not translate into a more favorable overall binding thermodynamics. The energy-decomposition results in Figure 8 further support this interpretation. For the three CDs, β-CD shows the most favorable LJ interactions with PFOA in both orientations, consistent with the fact that the cavity size of β-CD is the most suitable for PFAS binding.

**Figure 7:**
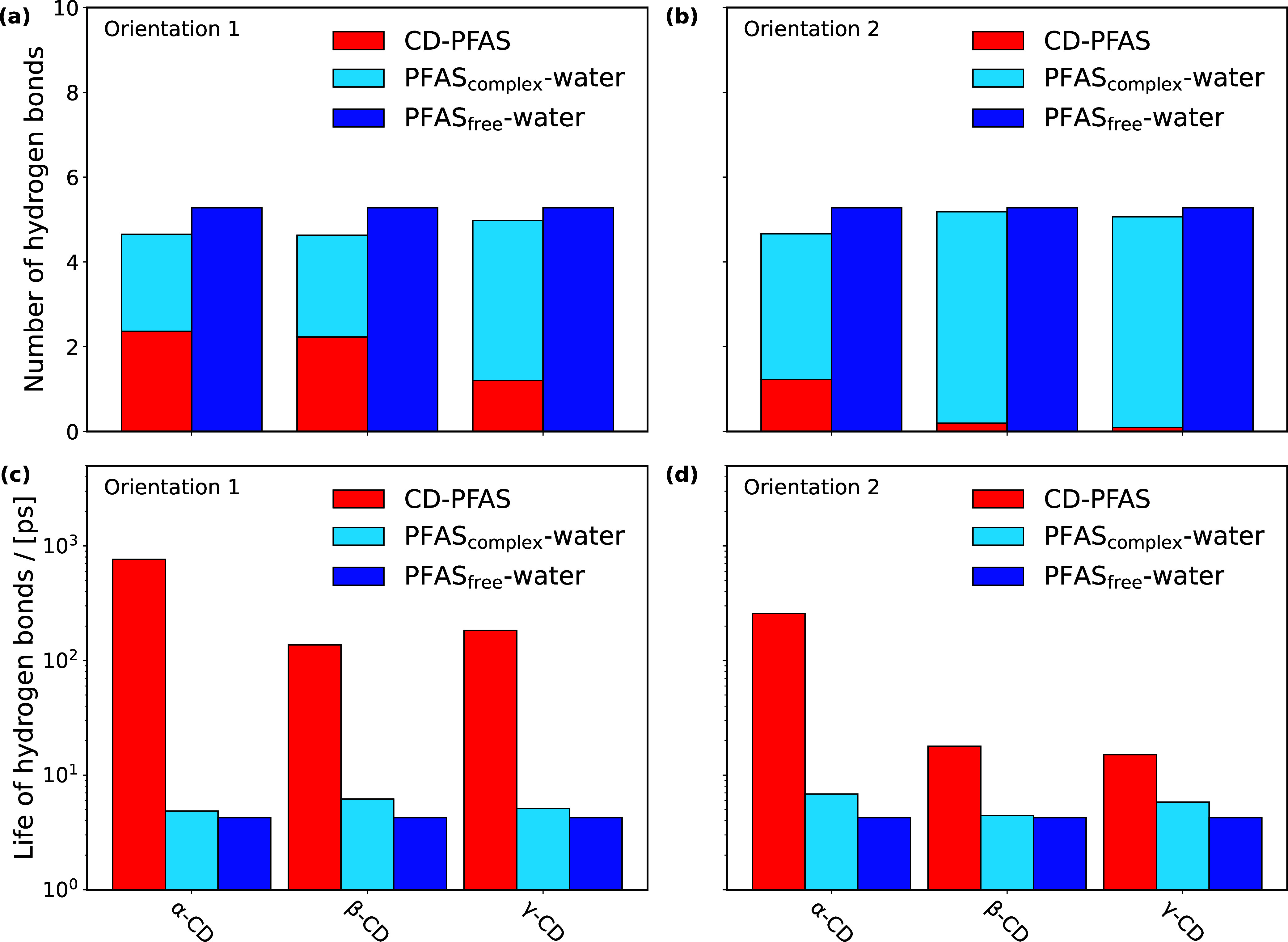
Number (a, b) and lifetime (c, d) of hydrogen bonds in α-, β-, and γ-CD–PFOA inclusion complexes for Orientation 1 (a, c) and Orientation 2 (b, d). PFAS_complex_ denotes PFOA in the inclusion complex and PFAS_free_ denotes free PFOA in pure aqueous solution.

**Figure 8:**
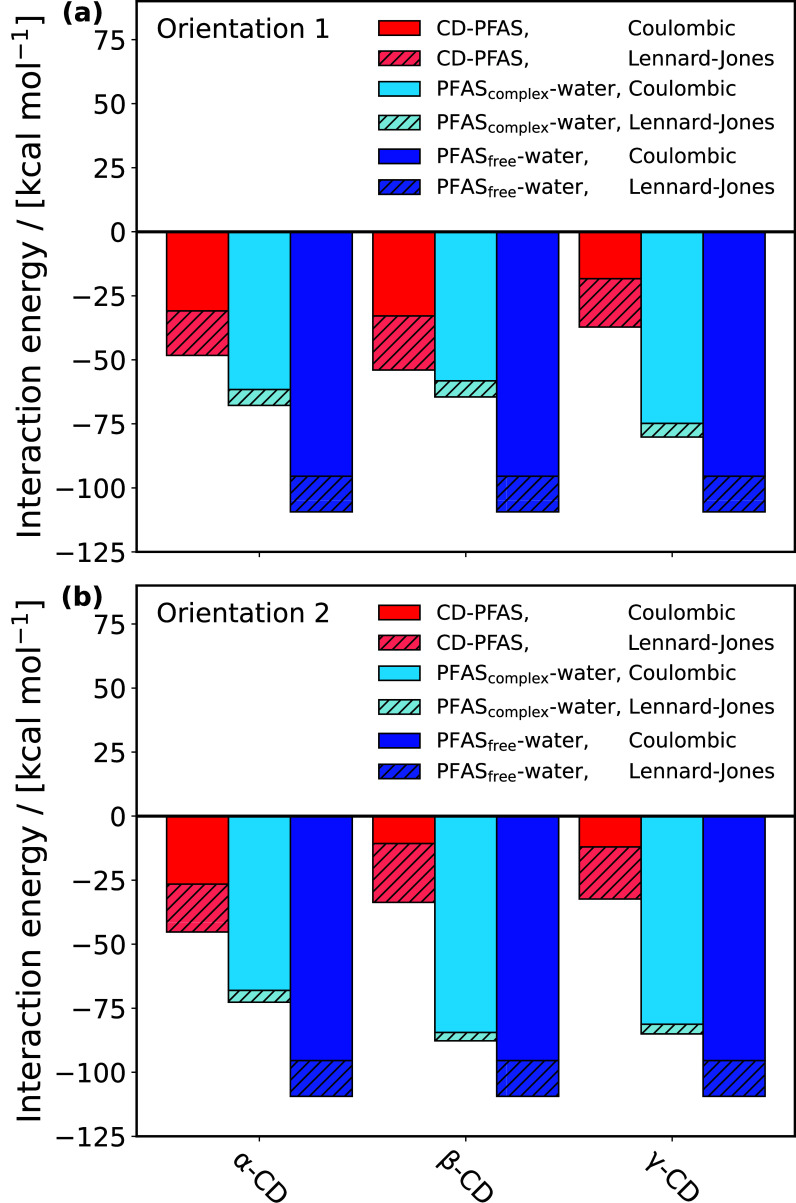
Energetic contributions in α-, β-, and γ-CD and PFOA systems for (a) Orientation 1 and (b) Orientation 2, where PFAS_complex_ denotes PFOA in the inclusion complex and PFAS_free_ denotes free PFOA in pure aqueous solution.

### Effect of Ionic Strength on Host–Guest Binding

To better understand ionic effects on the host–guest binding between β-CD and PFAS, and because dissolved salts are important constituents of many water-treatment streams, we simulated the β-CD–PFOA system in NaCl solutions with molalities ranging from 0 to 3 mol kg^–1^. In addition to simulations, we carried out experiments at 0, 1, and 2 mol kg^–1^ (the 3 mol kg^–1^ system was not measured experimentally because, under such high-salinity conditions, the samples did not dissolve sufficiently well on the time scale required for sample preparation). As shown in Figure 9, the experiments indicate a positive dependence of Δ*G* on salt concentration, i.e., increasing salinity increases Δ*G* and weakens β-CD–PFOA binding. In contrast, the simulations show an overall negative dependence regardless of the ion force field used or the binding orientation, with stronger binding predicted at higher salt concentration. This discrepancy should be interpreted with caution. As discussed in the Introduction, increasing cation concentration can in principle enhance the adsorption of long-chain anionic PFAS by screening electrostatic repulsion involving the anionic headgroups and thereby promoting closer hydrophobic contact between the fluorocarbon tail and the adsorbent surface.^5^ From this perspective, a salt-enhanced binding trend in the simulations is not necessarily unphysical. The three ion force fields also differ in the magnitude of the salt dependence. The JC model shows a clear decrease in Δ*G* beyond statistical uncertainty, whereas the two Li/Merz parameter sets show only a slight decrease that is close to the uncertainty range, indicating a much weaker concentration dependence. This comparison suggests that Li/Merz may be more reliable for describing salt-concentration effects in CD–PFAS binding. In addition, for Orientation 2 with Li/Merz-HFE, we observe that Δ*G* increases from 1 to 2 mol kg^–1^ by an amount that exceeds the statistical uncertainty. This may indicate that Li/Merz-HFE captures some energetic details of salt effects, possibly because its parameters were fitted to hydration energetics of ions. The ion force fields used here were parametrized for TIP3P water, and no ion force field has yet been specifically reparameterized for host–guest binding with Bind3P. Therefore, force field-related uncertainties are not negligible and may contribute to the deviation from experimental trends. Beyond simulation-side uncertainties, experimental uncertainties may also exist. While ITC measurements in pure water are reliable, we are not aware of strict benchmark studies that establish comparable accuracy in complex saline media. In addition, preparing PFAS solutions becomes increasingly difficult as salt concentration increases, potentially due to salting-out effects, which can introduce extra uncertainty into the measurements. Although the PFAS concentrations used here are well below the reported CMC values discussed in the Methods section, increasing salinity can still raise the likelihood of PFAS aggregation or ion-associated clustering. Such species may reduce the fraction of PFAS available to enter the CD cavity. Erdös et al.^78^ reported that negatively charged guests can show a degree of accumulation near the secondary opening of native β-CD. If an anionic PFAS must approach and enter the cavity through that wider rim, such interfacial crowding could in principle also hinder complex formation to some extent. Overall, the trend from simulations may therefore not be inherently incorrect from a mechanistic standpoint; however, because directly comparable literature data for PFAS–CD binding under saline conditions remain scarce, it is difficult at present to draw a definitive conclusion about the actual salt dependence.

**Figure 9:**
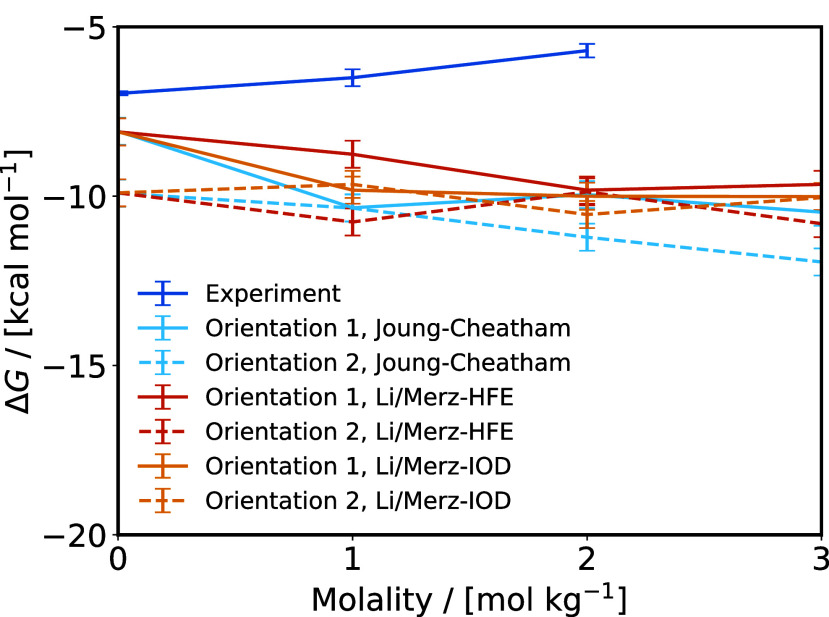
Host–guest binding free energies for the β-CD–PFOA system for both binding orientations under different NaCl molalities, including experimental values and simulation results obtained with three ion force fields (Joung-Cheatham, Li/Merz-HFE, and Li/Merz-IOD).^98,99^

To explain the observed dependence of binding free energy on NaCl concentration, we computed hydrogen bonds and energy decompositions. As shown in Figure 10, the number of CD–PFAS H-bonds changes only weakly with ionic strength. For Orientation 1, the number of PFOA–water H-bonds also changes only slightly, because the hydrophilic headgroup primarily forms H-bonds with primary hydroxyl groups of CD and has limited contact with external water. This is consistent with the smaller primary-side opening discussed above, which already limits water access to PFAS. Therefore, the introduction of ions has only a comparatively small additional effect on PFAS–water interactions in Orientation 1. For Orientation 2, where the PFOA headgroup is more exposed to water at the wider opening, the number of PFOA–water H-bonds decreases with increasing NaCl molality. In this configuration, Na^+^ ions have a stronger tendency than in Orientation 1 to occupy the region near the PFOA carboxylate group, and partially replace nearby water molecules, thereby reducing PFAS–water interactions. Consistent with these trends, Figure 11 shows that ionic strength has only a minor effect on direct CD–PFOA interaction energies, while Coulombic interactions involving PFOA (both in the complex and in bulk water) are substantially weakened in the presence of ions.

**Figure 10:**
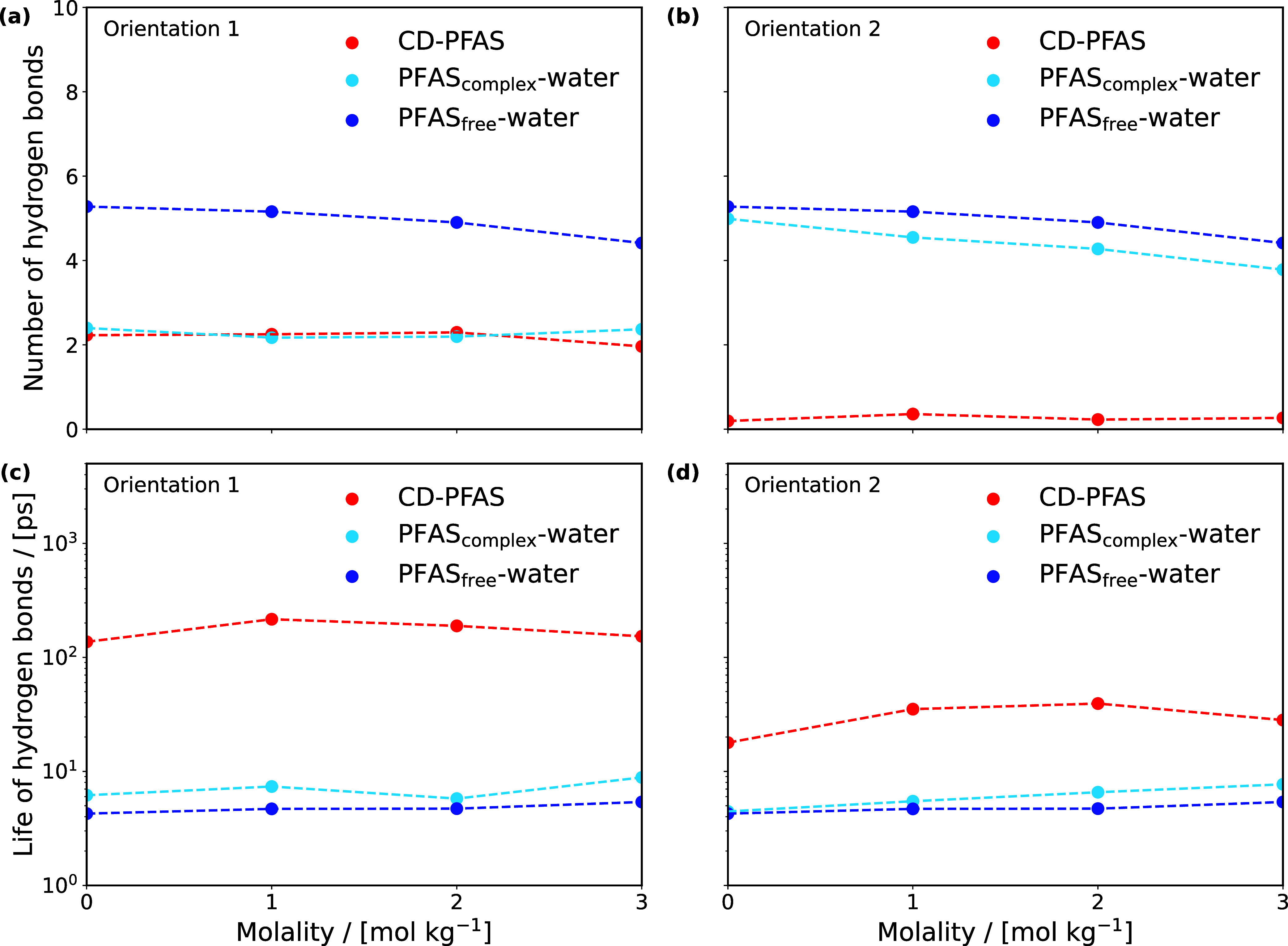
Number (a, b) and lifetime (c, d) of hydrogen bonds in β-CD–PFOA inclusion complexes under different NaCl molalities for Orientation 1 (a, c) and Orientation 2 (b, d). PFAS_complex_ denotes PFOA in the inclusion complex and PFAS_free_ denotes free PFOA in pure aqueous solution.

**Figure 11:**
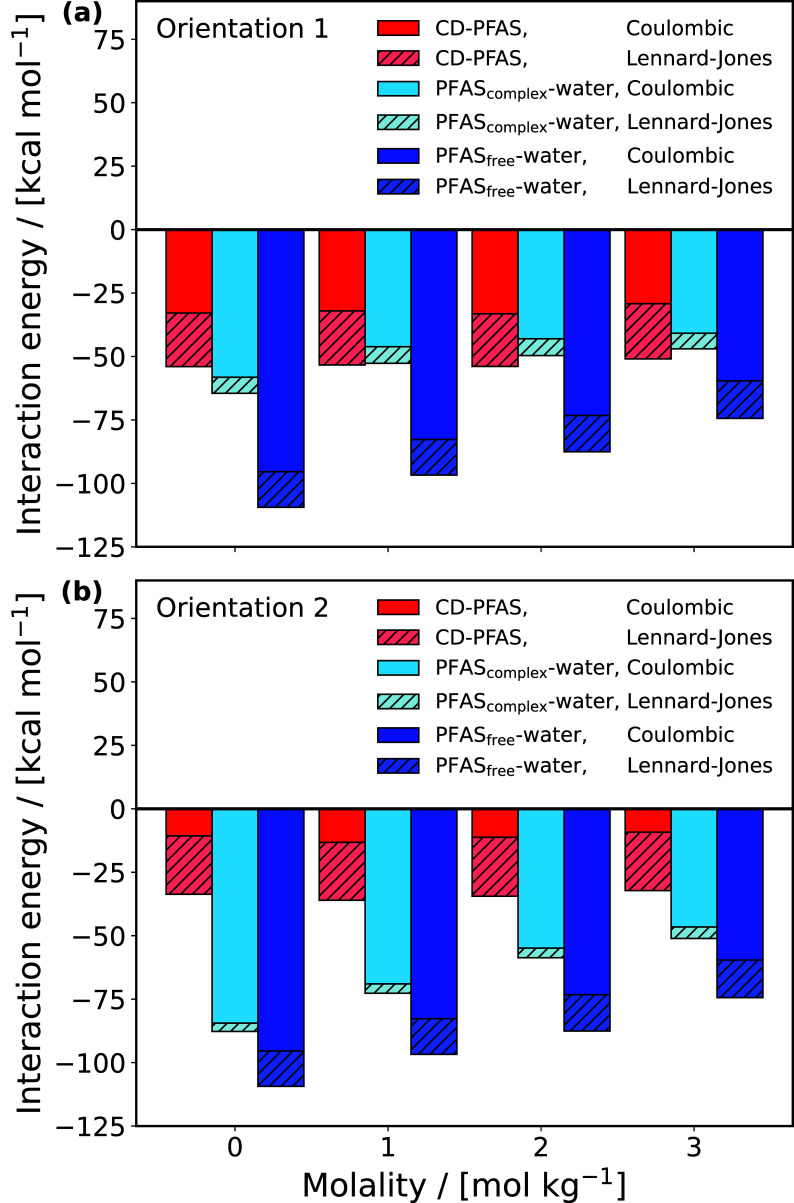
Energetic contributions in β-CD and PFOA system for (a) Orientation 1 and (b) Orientation 2 for different NaCl molalities, where PFAS_complex_ denotes PFOA in the inclusion complex and PFAS_free_ denotes free PFOA in pure aqueous solution.

Figure 12 provides additional insight into the free energy barriers in the β-CD–PFAS complex. Consistent with the analysis above, at the primary opening (Orientation 1), PFAS forms more H-bonds with CD; therefore, once salt is present, the pulling profiles (solid lines) show an almost concentration-independent barrier contribution. In contrast, at the larger secondary opening (Orientation 2), the charged headgroup of PFAS is less deeply embedded in the CD cavity and remains more exposed to the external solution, so the barrier increases progressively with increasing salt concentration.

**Figure 12:**
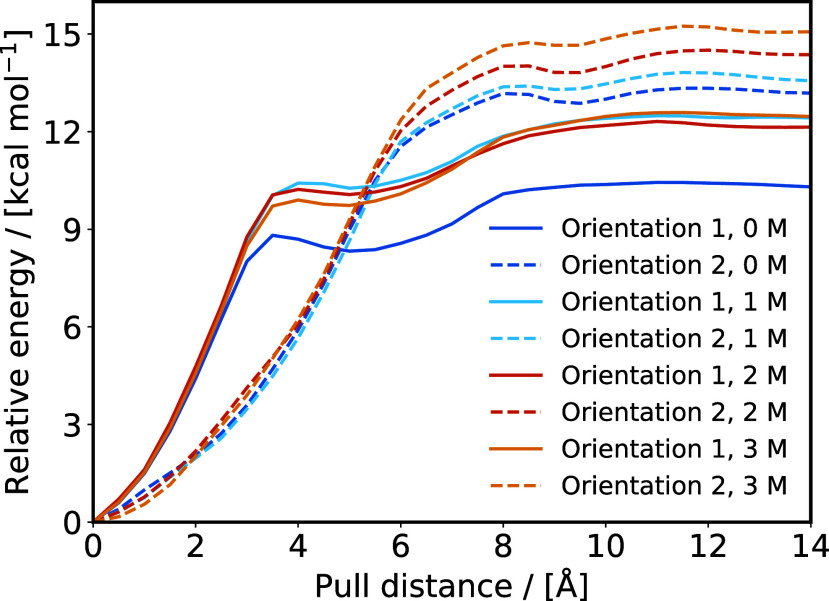
Free energy profiles in β-CD and PFOA system for Orientation 1 and 2 for different NaCl molalities.

Consistent with the analysis above, at the primary opening, PFAS forms relatively more H-bonds with CD; therefore, once salt is present, the pulling profiles (solid lines in Figure 12) show an almost concentration-independent barrier contribution. By contrast, at the larger secondary opening, the charged headgroup is less deeply embedded in the CD cavity and remains more exposed to the external solution, so the overall barrier increases progressively with increasing salt concentration.

### Effects of Functional Groups on the Host–Guest Binding for β-CD

To investigate how linker-like modification of cyclodextrin affects PFAS binding, we examined the binding of PFOA to β-CD derivatives containing different numbers of phenyl linkers. The binding free energies obtained with the implicit and explicit solvent models are shown in Figure 13. Because relevant experimental data are still lacking for these linker-modified systems, no experimental values are included in the figure for comparison. As discussed above, GAFF may introduce some uncertainty in the absolute description of PFAS host–guest binding, the present analysis focuses on a lateral comparison across systems with different numbers of linkers. Therefore, the variation of binding free energy with linker number mainly reflects how well each solvent model captures the effect of the linker environment. Because the added phenyl groups are generally described reasonably well by GAFF, the explicit solvent calculations recover the expected trend of increasingly favorable binding with increasing linker number, consistent with previous studies showing that aromatic or hydrophobic environments can strengthen PFAS association and confinement.^69,122–124^ The implicit solvent model fails to reproduce this trend and instead predicts progressively weaker binding as more linkers are introduced. A plausible explanation is that, once the newly added phenyl groups are described relatively well by the force field, the remaining errors of the solvent treatment become more apparent. In other words, the error cancellation discussed above becomes less effective for the implicit solvent model, so its description of linker-induced stabilization is poorer than that of the explicit solvent model.

**Figure 13:**
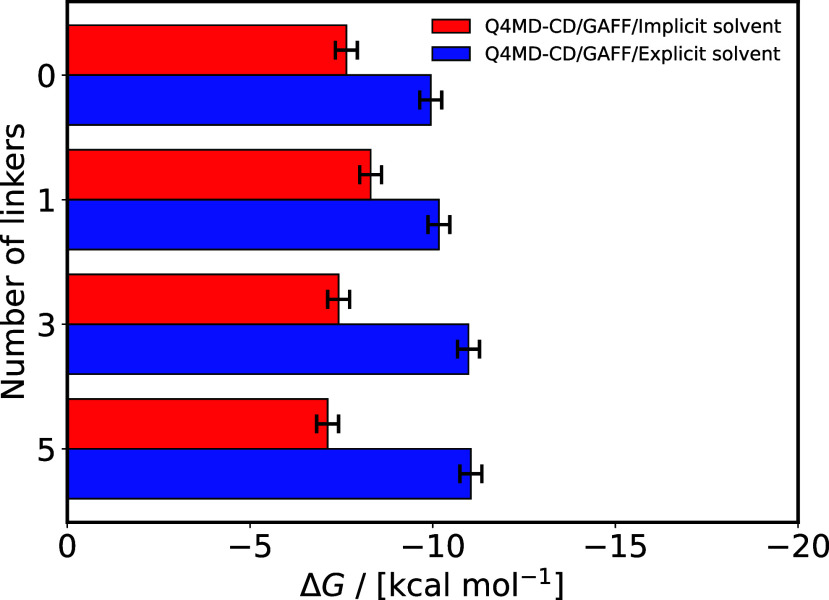
Host–guest binding free energies in β-CD and PFOA systems with different numbers of phenyl linkers. The apparent free energies are used here (eq 1).

To further understand how the linkers affect host–guest binding, we analyzed hydrogen bonding and energy decomposition. Figure 14 shows that the number of CD–PFOA hydrogen bonds changes only slightly with linker number for both orientations. For Orientation 2, the total number of hydrogen bonds shows a slight decrease as the number of linkers increases, suggesting that the linkers may weakly screen interactions between water and the carboxylate group located near the secondary opening. In terms of hydrogen-bond lifetimes, both orientations show some increase when the number of linkers reaches three. This may arise from the confining effect of the linkers, which can reduce PFAS mobility and thereby prolong the lifetime of CD–PFAS hydrogen bonds. Further insight is provided by the energy decomposition in Figure 15. In addition to the same decomposition used above, we further partitioned the cyclodextrin host into the cyclodextrin backbone and the linker groups. Here, Linker_Primary_ and Linker_Secondary_ denote the linker groups attached at the primary and secondary hydroxyl positions, respectively, whereas the remaining part of the host after removing all linker groups is denoted as CD_Backbone_. For Orientation 1, the direct CD–PFAS interaction energies do not change markedly with increasing linker number. For Orientation 2, however, the interaction between CD and PFAS becomes increasingly favorable as more linkers are introduced. At the same time, the LJ interaction between PFAS and water decreases, which is consistent with enhanced shielding of the guest from the solvent by the linkers. As the number of linkers increases, the LJ interactions between the CD backbone and PFAS and between the linkers and PFAS change only modestly, suggesting that the overall CD–PFAS binding geometry is not strongly altered by linker addition. The overall Coulombic attraction becomes weaker as linkers are introduced, as can be seen from Figure 15b. An explanation is that part of the positive charge is redistributed onto the linker-containing host, thereby reducing the effective positive charge on the cyclodextrin backbone and weakening its Coulombic attraction to PFOA. This effect has only a limited influence on Orientation 2, as discussed above, relying less on direct CD–PFAS hydrogen bonding and Coulombic stabilization. Finally, Figure 15c and 15f show the interaction energies between individual linkers and PFAS. These results indicate that the linker located on the side toward which the negatively charged PFAS headgroup points tends to form the stronger Coulombic interaction with the guest.

**Figure 14:**
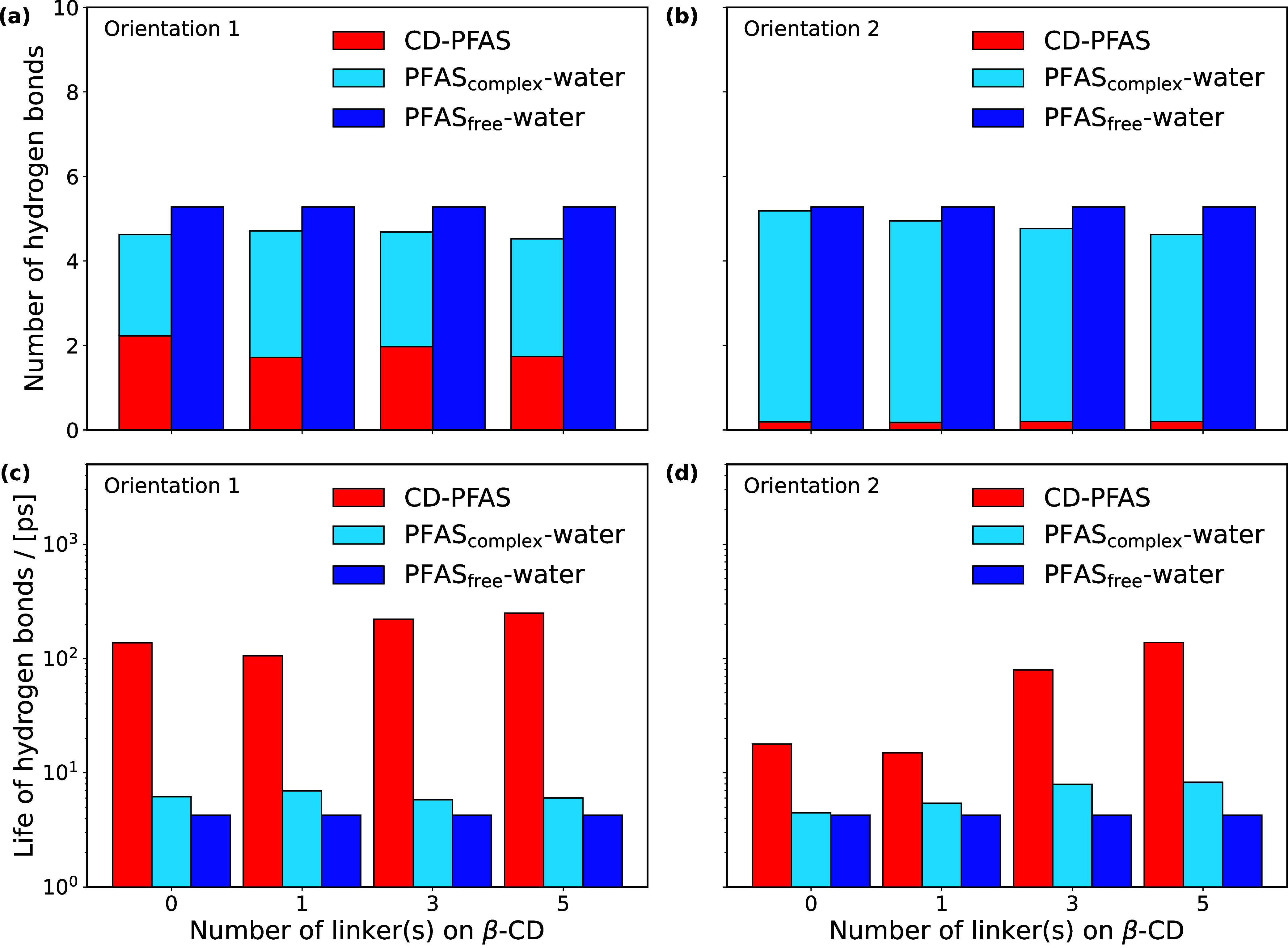
Number (a, b) and lifetime (c, d) of hydrogen bonds in β-CD and PFOA systems with different numbers of phenyl linkers for Orientation 1 (a, c) and Orientation 2 (b, d), where PFAS_complex_ denotes PFOA in the inclusion complex and PFAS_free_ denotes free PFOA in pure aqueous solution.

**Figure 15:**
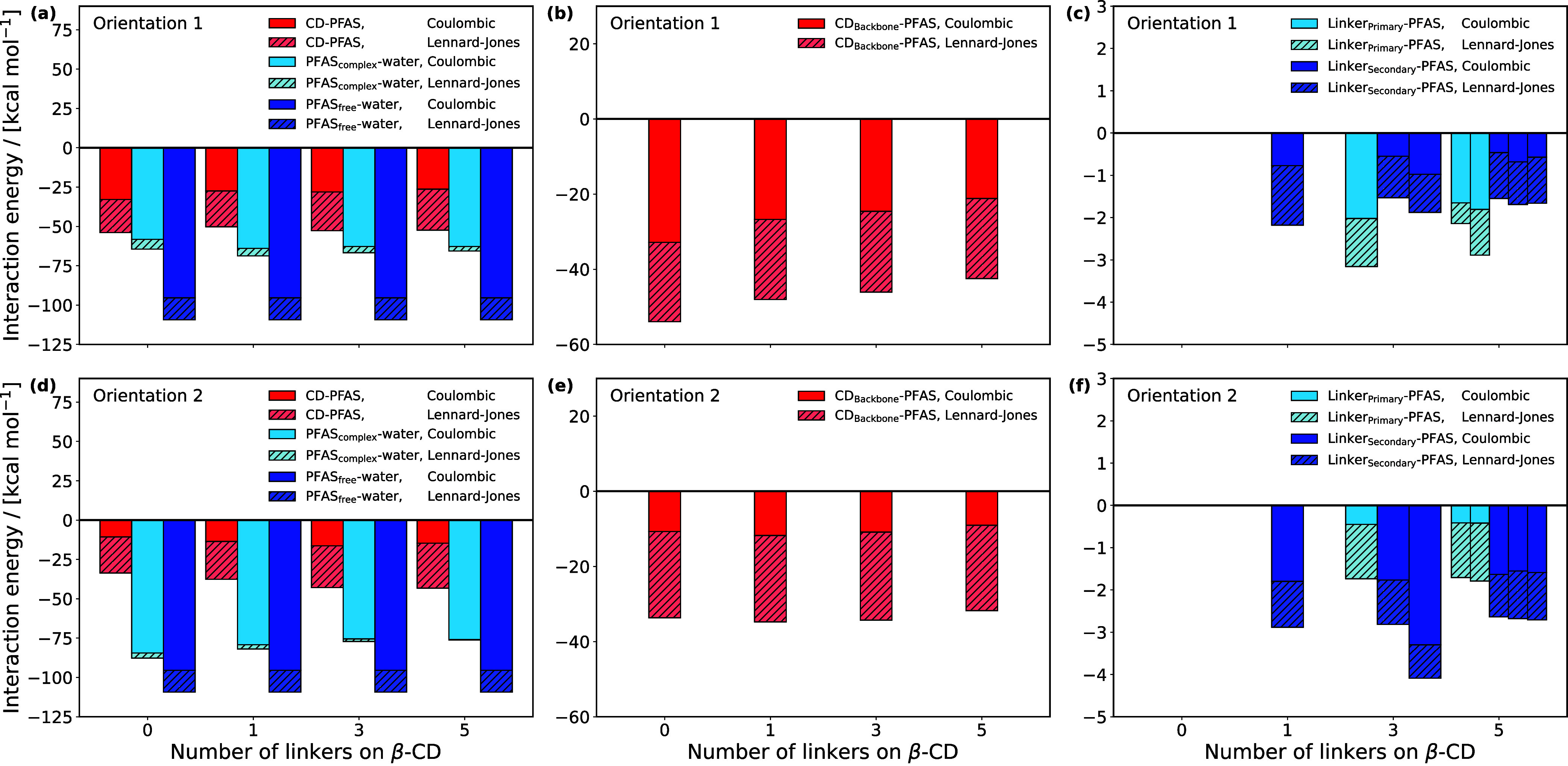
Energetic contributions in β-CD and PFOA system for Orientation 1 (a–c) and Orientation 2 (d–f) with different numbers of phenyl linkers, where PFAS_complex_ denotes PFOA in the inclusion complex, PFAS_free_ denotes free PFOA in pure aqueous solution, Linker_Primary_ and Linker_Secondary_ denote the linkers at the primary and secondary sides, respectively, and CD_Backbone_ denotes the cyclodextrin backbone after removing all phenyl groups.

## Conclusions

In this work, we combined ITC and force field-based MD simulations with the APR method to quantify the host–guest thermodynamics of seven linear PFAS with native α-, β-, and γ-CDs in water, and to assess how linker-like functionalization alters binding. For monomeric CD–PFAS complexes, the computed binding free energies obtained with both implicit and explicit solvent simulations are in reasonable agreement with our ITC measurements and with available literature data. Although simulations with the implicit solvent model yield closer agreement with experimental data for the β-CD–PFAS systems, this is not the case for the full native CD–PFAS data set and thus should not be interpreted as evidence of a more precise solvent description. Instead, the apparent agreement likely reflects compensation between the approximate solvation treatment and force field and/or host–guest cross-interaction errors for fluorinated guests. Once linker-modified CDs are considered, the implicit solvent model predicts a trend opposite to that observed for CD-based polymer systems, in which linker-like hydrophobic groups generally enhance small molecule adsorption. This contrast suggests that the explicit solvent model, which is built on a more realistic physical description of the solvent, may be more suitable for simulations of CD–guest complexation for a broad range of conditions and chemical species.

For the native hosts, β-CD consistently exhibits the strongest binding, whereas α-CD shows negligible affinity and γ-CD binds more weakly than β-CD. This difference is consistent with cavity-size matching and is further supported by interaction-energy decomposition, which shows that favorable molecular sizes and hydrophobic dehydration effects dominate the inclusion process. Consistently, the binding free energy correlates strongly with the solvent-accessible surface area change upon complexation (ΔSASA), highlighting the role of hydrophobic contributions. Hydrogen-bond analyses indicate that host–guest hydrogen bonding is not uniquely predictive of affinity. Rather, hydrogen-bond rearrangements primarily accompany dehydration and orientation-dependent complex formation.

To mimic the ions present in realistic water matrices and the local microenvironment surrounding cyclodextrin units in real polymers, we further examined how background ion concentration and linker modification affect β-CD–PFAS binding. Using explicit solvent simulations, we compared three ion parameter sets, JC, LM-HFE, and LM-IOD, and found that with increasing molality, the two Li/Merz parametrizations predict a smaller decrease in binding free energy for β-CD–PFAS than the JC model. We also investigated the binding free energies of linker-modified β-CD hosts and found that Bind3P water correctly reproduces the experimentally reported enhancement of PFAS adsorption with increasing linker number, supporting its applicability to more complex linker-functionalized cyclodextrin models. Although the implicit solvent model fails to capture this trend for different numbers of linkers, it remains useful for rapidly screening large numbers of host–guest systems and for generating data sets for machine-learning applications, because for calculations reaching the same level of statistical uncertainty, the implicit solvent model is more than an order of magnitude faster than the explicit solvent model. In addition, through energy decomposition, we could study the interactions formed between PFAS and individual linker groups. Specifically, the linker group positioned on the side toward which the negatively charged PFAS headgroup points forms a stronger Coulombic interaction with the guest than linkers on the opposite side. Meanwhile, increasing linker number progressively shields the guest from bulk solvent, as evidenced by the decreasing Lennard-Jones interaction between PFAS and water. Together, these results provide a microscopic explanation for why linker motifs in polymers can enhance PFAS uptake by cyclodextrin, as the linkers simultaneously strengthen direct electrostatic attraction to the PFAS headgroup and reduce the energetic cost of desolvating the guest.

Overall, our results provide molecular-level thermodynamic and mechanistic insight into CD–PFAS inclusion, which is a key step underlying PFAS capture by CD-based polymers. By examining the main building blocks of cyclodextrin polymers, including different native CDs and linker-modified β-CD models, and by considering water matrices containing different ion concentrations, this study offers molecular-scale insight relevant to both the development of new cyclodextrin polymers and their application under diverse conditions. These findings can inform the rational selection of CD cavity size and local hydrophobic environments in CD-based adsorbents, and they lay the groundwork for the next step of in silico construction of cyclodextrin polymer models and molecular simulation studies of PFAS adsorption by these materials across different conditions.

## Supplementary Material





## Notes

Scripts and force field parameter files in AMBER format for PFOA and β-CD system (ZIP)
Tables S1–S3: ITC measurement results for α-, β- and γ-CD with selected PFAS, binding free energies from simulations in Orientation 1 and 2 for α-, β-, and γ-CD with selected PFAS, and binding free energies from simulations in Orientation 1 and 2 for β-CD with TCP and HEX (PDF)
