# Supplementary material for: Inclusion Complexation of Native and Functionalized α‑, β‑, and γ‑Cyclodextrins with PFAS: An Experimental and Molecular Simulation Study
Source: J Phys Chem B. 2026 Jul 18;130(30):7718–37. doi: 10.1021/acs.jpcb.6c02825 (PMC13430634; doi:10.1021/acs.jpcb.6c02825)
Supplement: Supplementary file 2 [file jp6c02825_si_002.pdf]

Supporting Information for: Inclusion Complexation of Native and Functionalized α-, β-, and γ-Cyclodextrins with PFAS: An Experimental and Molecular Simulation Study

Bowen Sha,† Akhilesh Soodan,‡ Kim M. Lompe,‡ Gokhan Barin,¶ Thijs J.H. Vlugt,† Loukas D. Peristeras,§ and Othonas A. Moultos*,†

†*Engineering Thermodynamics, Process & Energy Department, Faculty of Mechanical Engineering, Delft University of Technology, Leeghwaterstraat 39, 2628 CB Delft, the Netherlands*

‡*Sanitary Engineering, Water Management Department, Faculty of Civil Engineering and Geosciences, Delft University of Technology, Stevinweg 1, 2628 CN Delft, the Netherlands*

¶*Cyclopure, Inc., Evanston, IL 60201, USA*

§*Molecular Thermodynamics and Modeling of Materials Laboratory, Institute of Nanoscience and Nanotechnology, National Center for Scientific Research "Demokritos", GR-15310 Aghia Paraskevi, Attikis, Greece*

E-mail: O.Moultos@tudelft.nl

Supporting Information Available

Attach–Pull–Release Method

The attach–pull–release (APR) method by Henriksen et al.[1] for host–guest binding calculations decomposes the standard binding free energy into three sequential steps. In the attachment phase, harmonic restraints on the guest's translational, rotational, and host conformational degrees of freedom are gradually turned on to define a well-controlled pulling coordinate. During the pulling phase, the same host-guest distance restraint is applied across all windows, with its target distance progressively increased to separate the two species until they no longer interact. In the release phase, the conformational restraints of host are removed while the guest is kept far away, and the release of guest restraints is performed analytically (i.e., no statistical uncertainty associated) with a standard-state correction to recover the unbound state. Free energies along this pathway are obtained by the multistate Bennett acceptance ratio (MBAR) estimator,[2] evaluated from the set of independent simulation windows.

In our study, the attachment phase comprised 17 windows. During these windows, the distance restraints on the guest were gradually changed, starting from 0, ending up to a force constant of 10.0 kcal mol^{-1} $Å^{-2}$, while the angle and torsion restraints reached 100.0 kcal mol^{-1} rad^{-2}. To prevent full dissociation of the host–guest complex, which can occur at very low λ values, flatwell restraints,[3] i.e., restraints with a flat-bottomed potential that exert no force within a predefined distance range and only become active when the restraint coordinate moves outside that range, with a constant of 10.0 kcal mol^{-1} $Å^{-2}$ were applied throughout. According to Henriksen et al., β-CD has substantial conformational flexibility. During pulling-stage simulations, the CD can stay in the distorted configuration that persists for several nanoseconds. The resulting fluctuations and conformational trapping lead to convergence difficulties of the host-guest binding calculations. To overcome this issue, 14 torsional restraints (two per glucopyranoside monomer) were imposed on the

β-CD backbone during the attachment stage of the free-energy calculation. Specifically, each monomer was restrained via two torsion angles: $O5_n$–$C1_n$–$O1_n$–$C4_{n+1}$ and $C1_n$–$O1_n$–$C4_{n+1}$–$C5_{n+1}$, with target values of 108.7° and −112.5°, respectively, and a force constant of 6.0 kcal mol^{-1} rad^{-2}. The pulling phase consisted of 29 windows in which the guest distance restraint was incrementally shifted from 6.0 Å to 20.0 Å in steps of 0.5 Å.

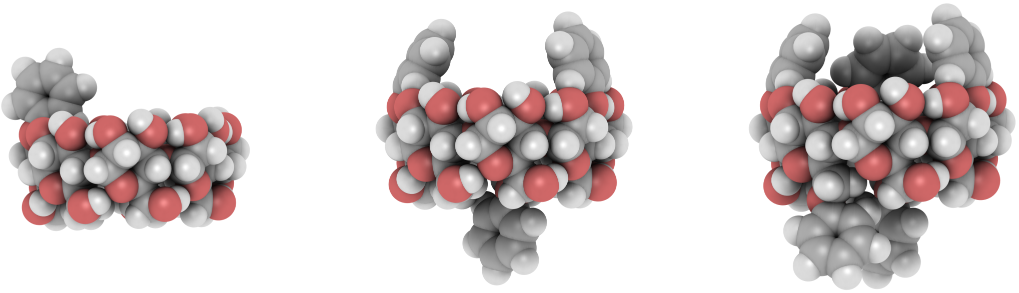

Figure S1: 3D structures of linker-modified β-CDs, from left to right: β-CDs modified with 1, 3, and 5 linkers, respectively.

Host–guest binding thermodynamic data obtained from isothermal titration calorimetry and the molecular simulations using the APR method

Table S1a: ITC thermodynamic parameters for β-CD binding with selected PFAS. Values obtained from fitting integrated heats to a $K_{1:1}$ model.

PFAS	Cell conc. (mM)	K (M^{-1})	ΔH (kcal mol^{-1})	ΔG (kcal mol^{-1})	ΔS (cal mol^{-1} K^{-1})	c-value
PFBA	0.94	$(1.25 \pm 0.6) \times 10^3$	-0.018 ± 0.02	-4.22 ± 0.28	12.7	1.7
PFHxA	0.64	$(1.27 \pm 0.03) \times 10^4$	-4.11 ± 0.03	-5.6 ± 0.03	4.99	8.1
PFOA	0.48	$(1.28 \pm 0.12) \times 10^5$	-5.32 ± 0.07	-6.96 ± 0.06	5.54	35
PFBS	0.67	$(1.04 \pm 0.02) \times 10^4$	-5.46 ± 0.01	-5.48 ± 0.01	0.07	8
PFHxS	0.50	$(1.73 \pm 0.02) \times 10^5$	-6.42 ± 0.04	-7.14 ± 0.01	2.42	89
PFOS	0.40	$(6.17 \pm 3.32) \times 10^3$	-32.1 ± 49.3	-5.17 ± 0.02	-90.3	2.5
PFNA	0.40	$(5.01 \pm 1.33) \times 10^4$	-7.72 ± 0.30	-6.41 ± 0.32	-4.37	15

Table S1b: ITC thermodynamic parameters for α-CD binding with selected PFAS. Values obtained from fitting integrated heats to a $K_{1:1}$ model.

PFAS	Cell conc. (mM)	K (M^{-1})	ΔH (kcal mol^{-1})	ΔG (kcal mol^{-1})	ΔS (cal mol^{-1} K^{-1})	c-value
PFBA	0.94	$(3.03 \pm 6.07) \times 10^3$	-0.11 ± 2.3	-4.75 ± 1.19	15.6	2.8
PFHxA	0.64	3.39 ± 139	$(-0.13 \pm 0.5) \times 10^3$	≈ -2.94	-39.5	< 1
PFOA	0.48	$(0.3 \pm 3) \times 10^4$	$(-2.2 \pm 7.8) \times 10^3$	-4.7 ± 5.9	8.59	1.5
PFBS	0.67	208 ± 24.1	-0.58 ± 0.05	-3.16 ± 0.07	8.6	< 1
PFHxS	0.50	930 ± 45.6	0.64 ± 0.02	-4.04 ± 0.02	15.7	< 1

Table S1c: ITC thermodynamic parameters for γ-CD binding with selected PFAS. Values obtained from fitting integrated heats to a $K_{1:1}$ model.

PFAS	Cell conc. (mM)	K (M^{-1})	ΔH (kcal mol^{-1})	ΔG (kcal mol^{-1})	ΔS (cal mol^{-1} K^{-1})	c-value
PFBA	0.94	$(0.1 \pm 3) \times 10^3$	$(0.001 \pm 3.2) \times 10^5$	-2.7	38.6	< 1
PFHxA	0.64	725 ± 799	0.12 ± 0.05	-3.91 ± 0.05	13.5	< 1
PFOA	0.48	$(1.01 \pm 0.03) \times 10^3$	0.80 ± 0.01	-4.10 ± 0.02	16.4	< 1
PFBS	0.67	87.2 ± 24.5	-0.52 ± 0.13	-2.65 ± 0.17	7.14	< 1
PFHxS	0.50	$(1.56 \pm 0.14) \times 10^3$	0.24 ± 0.01	-4.35 ± 0.01	15.4	< 1
PFNA	0.40	$(1.32 \pm 0.25) \times 10^3$	21.6 ± 100.6	-4.24 ± 1.14	86.9	< 1

Table S2a: Binding free energies (ΔG, kcal mol^{-1}) of PFAS with α-, β-, and γ-cyclodextrin computed from MD simulations in Orientation 1. Im = implicit solvent; Ex = explicit solvent.

	α-CD		β-CD		γ-CD	
PFAS	Im	Ex	Im	Ex	Im	Ex
PFBA	2.79	−0.07	−1.90	−4.24	−1.73	−2.37
PFHxA	1.52	1.74	−5.23	−7.51	−3.70	−3.82
PFOA	0.58	2.05	−7.17	−8.08	−5.73	−4.73
PFBS	1.08	−1.12	−5.01	−6.90	−3.30	−3.79
PFHxS	2.39	1.58	−7.03	−9.33	−5.20	−5.28
PFOS	0.39	0.01	−8.15	−10.14	−6.92	−6.31
PFNA	—	—	−3.19	−7.91	—	—

Table S2b: Binding free energies (ΔG, kcal mol^{-1}) of PFAS with α-, β-, and γ-cyclodextrin computed with MD simulations in Orientation 2. Im = implicit solvent; Ex = explicit solvent.

	α-CD		β-CD		γ-CD	
PFAS	Im	Ex	Im	Ex	Im	Ex
PFBA	0.68	−2.95	−2.43	−4.69	−1.94	−2.73
PFHxA	−0.61	−5.27	−5.32	−8.06	−3.59	−4.53
PFOA	1.36	0.44	−7.28	−9.92	−5.68	−6.35
PFBS	−0.23	−3.44	−5.05	−7.43	−3.48	−3.96
PFHxS	−0.36	−0.54	−7.27	−8.53	−4.99	−5.85
PFOS	1.16	0.46	−10.71	−11.36	−6.58	−6.68
PFNA	—	—	−2.68	−11.24	—	—

Table S3: Binding free energies (ΔG, kcal mol^{-1}) of TCP[4,5] and HEX[1] with β-CD from MD simulations here and from the literature (explicit solvent simulations and experimental results). Im = implicit solvent; Ex = explicit solvent. Expt. = experiment.

Compound	Im	Ex	Literature Ex	Literature Expt.
TCP	-5.6 ± 0.3	-3.2 ± 0.4	-3.7 ± 0.4	-2.8
HEX	-1.8 ± 0.2	-3.5 ± 0.3	-3.9 ± 0.2	-2.3 ± 0.1

Description of Simulation Files and Pipeline for the β-CD–PFOA APR Binding Free Energy Calculations

The ZIP archive "Scripts_and_forcefields" contains the complete set of input structures, force field parameters, setup and analysis scripts, and AMBER input files required to compute APR binding free energies of the β-CD–PFOA complex in two orientations and two solvent models (implicit GB and explicit Bind3P). A summary of each file's purpose is given below; full details and step-by-step instructions are provided in the `README.md` file included in the archive.

- `BCD-FOA1.pdb` and `BCD-FOA2.pdb`: Starting complex structures for orientation 1 (C3 of PFOA leads during pulling) and orientation 2 (C7 leads).
- `BCD.mol2` and `FOA.mol2`: Tripos Mol2 topologies for β-CD and PFOA.
- `BCD.frcmod` and `FOA.frcmod`: AMBER force field modification files for the host and guest.
- `setup_nopbc1.py` and `setup_nopbc2.py`: Python scripts that build the vacuum complex, add dummy atoms, and generate APR restraint windows for implicit-solvent (GB) simulations.
- `setup_pbc1.py` and `setup_pbc2.py`: Python scripts that solvate each window with Bind3P water and add counterions for explicit-solvent simulations.
- `minimize_nopbc.in`, `production_nopbc1.in`, `production_nopbc2.in`: AMBER minimization, short equilibration, and production input files for GB runs.
- `minimize_pbc.in`, `production_pbc1.in`, `production_pbc2.in`, `production_pbc3.in`: AMBER minimization, stepwise equilibration, and production input files for explicit-solvent runs.

- `analysis_nopbc1.py`, `analysis_nopbc2.py`, `analysis_pbc1.py`, `analysis_pbc2.py`: Python scripts that perform thermodynamic integration analysis on the output trajectories and compute the binding free energy.

- `run.sh`: Main script that sequentially executes the full pipeline (setup, minimization, equilibration, production, and analysis).

- `BCD_1L.pdb`, `BCD_3L.pdb`, and `BCD_5L.pdb`: 3D structures of the 1-, 3-, and 5-substituted linker-modified β-CD hosts, respectively.

All simulations were performed using the `pmemd.cuda` engine of AMBER. The pipeline requires the `paprika`, `parmed`, `numpy`, and `matplotlib` Python packages. Detailed prerequisites, workflow description, and instructions for running individual stages are documented in the `README.md` file.

References

(1) Henriksen, N. M.; Fenley, A. T.; Gilson, M. K. Computational calorimetry: high-precision calculation of host–guest binding thermodynamics. *Journal of Chemical Theory and Computation* **2015**, *11*, 4377–4394.

(2) Shirts, M. R.; Chodera, J. D. Statistically optimal analysis of samples from multiple equilibrium states. *The Journal of Chemical Physics* **2008**, *129*.

(3) Clark, F.; Robb, G.; Cole, D. J.; Michel, J. Comparison of receptor–ligand restraint schemes for alchemical absolute binding free energy calculations. *Journal of Chemical Theory and Computation* **2023**, *19*, 3686–3704.

(4) Erdős, M.; Hartkamp, R.; Vlugt, T. J. H.; Moultos, O. A. Inclusion complexation of organic micropollutants with β-cyclodextrin. *Journal of Physical Chemistry B* **2020**, *124*, 1218–1228.

(5) Hanna, K.; de Brauer, C.; Germain, P. Solubilization of the neutral and charged forms of 2,4,6-trichlorophenol by β-cyclodextrin, methyl-β-cyclodextrin and hydroxypropyl-β-cyclodextrin in water. *Journal of Hazardous Materials* **2003**, *100*, 109–116.
